# Paleogenomic Evidence for Multi-generational Mixing between Neolithic Farmers and Mesolithic Hunter-Gatherers in the Lower Danube Basin

**DOI:** 10.1016/j.cub.2017.05.023

**Published:** 2017-06-19

**Authors:** Gloria González-Fortes, Eppie R. Jones, Emma Lightfoot, Clive Bonsall, Catalin Lazar, Aurora Grandal-d’Anglade, María Dolores Garralda, Labib Drak, Veronika Siska, Angela Simalcsik, Adina Boroneanţ, Juan Ramón Vidal Romaní, Marcos Vaqueiro Rodríguez, Pablo Arias, Ron Pinhasi, Andrea Manica, Michael Hofreiter

**Affiliations:** 1 Department of Life Sciences and Biotechnology, University of Ferrara, Via L. Borsari 46, Ferrara 44100, Italy; 2 Institute for Biochemistry and Biology, University of Potsdam, Karl-Liebknecht-Straße 24-25, 14476 Potsdam OT Golm, Germany; 3 Department of Zoology, University of Cambridge, Downing Street, Cambridge CB2 3EJ, UK; 4 McDonald Institute for Archaeological Research, University of Cambridge, Downing Street, Cambridge CB2 3ER, UK; 5 School of History, Classics and Archaeology, University of Edinburgh, William Robertson Wing, Old Medical School, Teviot Place, Edinburgh EH8 9AG, UK; 6 National History Museum of Romania, Bucharest 030026, Romania; 7 Instituto Universitario de Xeoloxía, Universidade da Coruña, A Coruña 15081, Spain; 8 Department of Zoology and Physical Anthropology, Complutense University of Madrid, Madrid 28040, Spain; 9 “Olga Necrasov” Centre for Anthropological Research of the Romanian Academy, Iaşi Branch, Theodor Codrescu Strada 2, 700481 Iaşi, Romania; 10 “Vasile Pârvan” Institute of Archaeology, Romanian Academy, Henri Coandă Strada 11, Bucharest 010667, Romania; 11 International Institute of Prehistorical Research, University of Cantabria-Government of Cantabria-Bank of Santander, Santander 39005, Spain; 12 School of Archaeology and Earth Institute, Belfield, University College Dublin, Dublin 4, Ireland; 13 Department of Anthropology, University of Vienna, Althanstrasse 14, 1090 Vienna, Austria

**Keywords:** ancient DNA, Eneolithic, Neolithic transition, Romania, Iron Gates, Mesolithic

## Abstract

The transition from hunting and gathering to farming involved profound cultural and technological changes. In Western and Central Europe, these changes occurred rapidly and synchronously after the arrival of early farmers of Anatolian origin [1, 2, 3], who largely replaced the local Mesolithic hunter-gatherers [1, 4, 5, 6]. Further east, in the Baltic region, the transition was gradual, with little or no genetic input from incoming farmers [7]. Here we use ancient DNA to investigate the relationship between hunter-gatherers and farmers in the Lower Danube basin, a geographically intermediate area that is characterized by a rapid Neolithic transition but also by the presence of archaeological evidence that points to cultural exchange, and thus possible admixture, between hunter-gatherers and farmers. We recovered four human paleogenomes (1.1× to 4.1× coverage) from Romania spanning a time transect between 8.8 thousand years ago (kya) and 5.4 kya and supplemented them with two Mesolithic genomes (1.7× and 5.3×) from Spain to provide further context on the genetic background of Mesolithic Europe. Our results show major Western hunter-gatherer (WHG) ancestry in a Romanian Eneolithic sample with a minor, but sizeable, contribution from Anatolian farmers, suggesting multiple admixture events between hunter-gatherers and farmers. Dietary stable-isotope analysis of this sample suggests a mixed terrestrial/aquatic diet. Our results provide support for complex interactions among hunter-gatherers and farmers in the Danube basin, demonstrating that in some regions, demic and cultural diffusion were not mutually exclusive, but merely the ends of a continuum for the process of Neolithization.

## Highlights

• Demic and cultural diffusions underlie the Neolithic period in the Danube basin
• A large WHG genome component was present in Eneolithic communities in this region
• The further east in Europe, the weaker the genetic component of Anatolian farmers
• Environmental factors may account for a demic diffusion breakdown in these regions

## Teaser

A key question in archaeological research is whether the transition from hunting and gathering was more reliant on the movement of people or ideas. González-Fortes et al. show, based on genomes of several ancient humans, that in parts of Romania, it was actually a mix of both processes that took place during this so-called Neolithization process.

## Results

**Figure 1:**
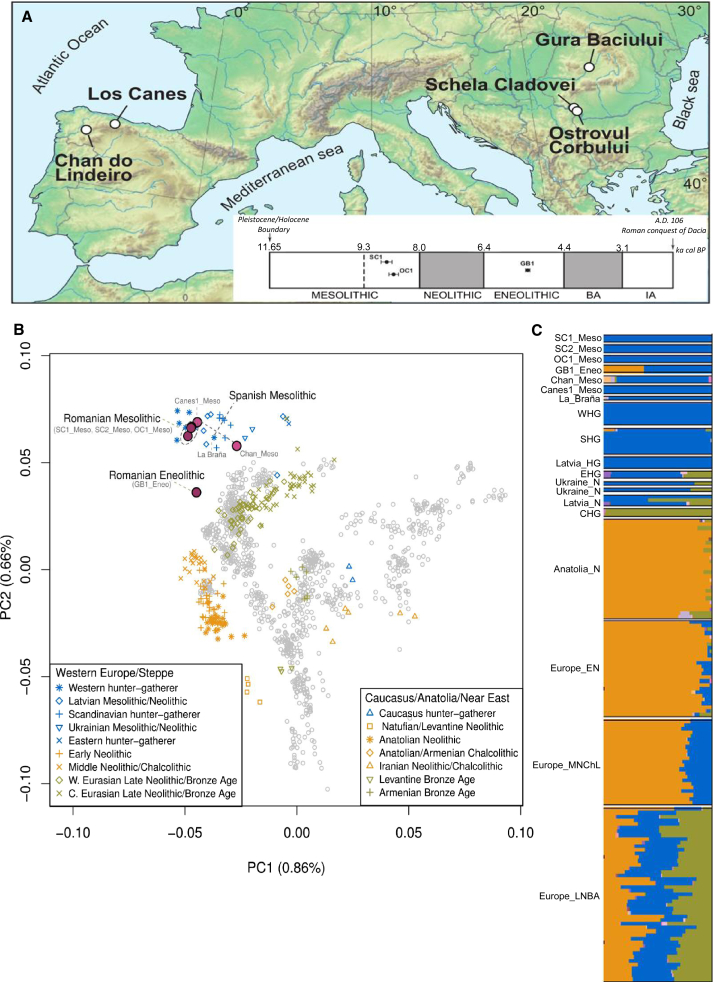
Geographical, Archaeological, and Genetic Information for the Ancient Spanish and Romanian Samples (A) Map showing the location of the archaeological sites: Chan do Lindeiro (Chan_Meso), Canes (Canes1_Meso), Schela Cladovei (SC1_Meso and SC2_Meso), Ostrovul Corbului (OC1_Meso), and Gura Baciului (GB1_Eneo). Gura Baciului is some 250 km north-northeast of the Iron Gates (Schela Cladovei and Ostrovul Corbului), on a small river that eventually connects with the Danube via the River Tisza. Along with the map we include a timeline with the radiocarbon dates of our samples and the time frame of the different prehistoric periods in Romania. (B) Principal-component analysis (PCA). Ancient data (Data S1) were projected onto the first two principal components defined by selected Eurasians from the Human Origins dataset [8, 9]. The Spanish (Chan_Meso and Canes1_Meso) and Romanian (SC1_Meso, SC2_Meso, and OC1_Meso) Mesolithic samples cluster close to European hunter-gatherer samples. The Eneolithic Romanian sample (GB_Eneo) locates in a different region of the plot, between European hunter-gatherer and farmer samples. (C) ADMIXTURE analysis. ADMIXTURE results are shown at *K* = 17. The Spanish and Romanian hunter-gatherer samples are composed entirely of the “blue” component, which is also found in other European hunter-gatherer samples, with the exception of the oldest Spanish Mesolithic sample, Chan_Meso, which also has a “lilac” component found in South Indians. The Eneolithic individual GB1_Eneo has the “blue” as well as the “orange” component that predominates in early European and Anatolian farmer samples. Error bars in (A) correspond to the radio carbon ages of samples SC1_Meso, OC1_Meso, and GB1_Eneo as reported in Table 1. See also Figures S1–S4 and Table S3.

We investigated the interactions between hunter-gatherers and Neolithic farmers in the Lower Danube basin in Romania by recovering the genomes of four prehistoric individuals: a Mesolithic hunter-gatherer from Ostrovul Corbului (OC1_Meso) dated at 8.7 thousand years ago (kya), two Mesolithic hunter-gatherers from Schela Cladovei (SC1_Meso and SC2_Meso) dated at around 8.8 kya, and an Eneolithic (the period between the Neolithic and the Bronze Age) individual dated at 5.3 kya from Gura Baciului (GB1_Eneo), located north-northeast of the Iron Gates on a terrace of the Suceag creek (Figure 1A and STAR Methods, Method Details). Contact between hunter-gatherers and farmers has been hypothesized for a number of archaeological sites across Europe. In 2012, Skoglund et al. [4] reported the first genomic data suggesting different origins for hunter-gatherers and early farmers in Scandinavia. Further work [1, 5, 6] provided additional paleogenomic evidence that Neolithization was driven by immigration of farming populations, supporting the demic diffusion model, at least for Scandinavia and Western and Central Europe. In Southeast Europe, the Lower Danube Basin has provided some of the best evidence for cultural exchange, and thus possible mixing, between hunter-gatherers and farmers [10, 11, 12]. Archaeological data put the arrival of the typical Neolithic package, including farming, pottery, and new burial practices, at around 8 kya. Isotopic analysis of very late Mesolithic burials from Lepenski Vir around that time revealed several individuals whose diets were relatively high in terrestrial proteins, a profile more typical of farming communities [11]; although the genetic origin of these individuals is unknown, their presence points to contact of this Mesolithic community with farmers (either through cultural exchange or immigration). The presence of personal ornaments of Neolithic type in some Late Mesolithic (8.3–8.0 kya) graves at Lepenski Vir and Vlasac [13] and the recovery of cereal starch granules from dental calculus on Mesolithic teeth from Vlasac [12] further support the hypothesized adoption of new practices by local hunter-gatherers in the Lower Danube basin [11].

### Laboratory and Next-Generation Sequencing Data Processing

We extracted DNA from petrous bone samples of each individual, constructed one double-stranded Illumina sequencing library from each extract, and sequenced the libraries on a HiSeq2000 platform. After mapping and filtering, endogenous content ranged from 23.7% to 55.2%, and genome coverage ranged from 1.1× to 5.3× (Table 1). All samples displayed characteristic ancient DNA (aDNA) damage patterns, with the average read length between 62 and 109 base pairs and deamination rates of 14% to 25% at the ends of the molecules (Figure S1). Contamination estimates, based on the number of non-consensus bases in the mitochondria and the X chromosome in males, were between 0.4% and 2.2% (Table S2).

**Table 1:** Origin, Age, Next-Generation Sequencing Data, Uniparental Haplogroups, and Stable Carbon and Nitrogen Isotope Values Related to Diet for the Samples Analyzed in This Study

Sample ID	Site	Cal BP Age Range (2σ) [OxCal 4.2]^a^	Genome Coverage	Biol. Sex	mtDNA hg.	Y hg.	Isotope Values (‰)^b^		
							δ^13^C	δ^15^N	C:N Ratio^c^
SC1_Meso	Schela Cladovei (Romania)	8,814 ± 261	1.11×	XY	U5b2c	R	−18.5	15.0	3.2
SC2_Meso	Schela Cladovei (Romania)	−	2.83×	XY	U5a1c	R1	−19.1	14.7	3.2
OC1_Meso	Ostrovul Corbului (Romania)	8,704 ± 269	1.86×	XY	K1 + 16362	R1b	−18.7	15.5	3.1
GB1_Eneo	Gura Baciului (Romania)	5,377 ± 77	4.05×	XX	K1a4a	NA	−20.0	12.7	3.3
Chan_Meso	Chan do Lindeiro (Spain)	9,131 ± 124	5.28×	XX	U5b	NA	−20.5	8.4	3.1
Canes1_Meso	Canes (Spain)	7,115 ± 130	1.73×	XX	U5a2a	NA	−20.0	7.9	−

Cal BP, calibrated age before present; hg., haplogroup. See also Figure S4 and Tables S1, S2, and S4.

a The radiocarbon dates of samples from the Iron Gates (Schela Cladovei and Ostrovul Corbului) were corrected for the Danube Freshwater Reservoir Effect [14].

b Isotope values for SC1_Meso, Canes1_Meso, and Chan_Meso were published by [14], [15], and [16], respectively. Isotope values for OC1_Meso, SC2_Meso, and GB1_Eneo were obtained in this study.

c The C:N atomic ratio serves as an indicator of collagen preservation suitable for radiocarbon dating and paleodiet reconstruction.

### Phenotypic Traits

We investigated a number of phenotypic traits in our ancient samples. All three Romanian Mesolithic individuals were predicted to have dark hair and brown eyes, whereas the Eneolithic individual was predicted to have dark hair and light eye pigmentation (Figure S2 and Table S3). Based on the presence of the ancestral forms of both *SLC45A2* (rs1426654) and *SLC24A5* (rs16891982), two genes that were found to have gone through strong positive selection for skin depigmentation in the ancestors of modern Europeans, the three Romanian Mesolithic individuals were predicted to have had dark skin pigmentation. The Eneolithic individual most likely had a lighter skin tone, as it was homozygous for the derived version of *SLC45A2* and heterozygous for the derived version of *SLC24A5*. Although an increase in the frequencies of these variants is generally associated with the Neolithic, it should be noted that they were already present at low frequency among Scandinavian hunter-gatherers [8], and a copy of the derived variant of *SLC45A2* was also present in our late Spanish Mesolithic sample, Canes1_Meso. All individuals investigated were unlikely to be able to digest lactose as adults, as they all carried ancestral alleles at two key positions in the gene *MCM6* (rs4988235 and rs182549).

### Mitochondrial and Y Chromosome Haplogroups

At the level of mitochondrial DNA sequences, SC1_Meso belongs to U5b (Table 1), the same subhaplogroup to which a number of Western hunter-gatherers (WHGs) belong [17, 18, 19, 20]. SC2_Meso was assigned to U5a, a subhaplogroup mainly found in Scandinavian [5, 21] and Latvian hunter-gatherers and also in some samples from later periods in Eastern and Central Europe [6, 7, 22]. The two other samples from Romania, the Mesolithic OC1_Meso and the Eneolithic GB1_Eneo, both belong to K1, a haplogroup commonly found among early European farmers [17, 18] (see Table S4 and Method Details in STAR Methods for description of haplogroup assignment). At the Y chromosome level, all of our three male samples, SC1_Meso, SC2_Meso, and OC1_Meso, were assigned to the R1 and R1b haplogroups, both common in modern Europeans (Table 1 and STAR Methods, Quantification and Statistical Analysis).

### Population Genomic Analysis

We investigated the genome-wide relationships of our samples to modern and other ancient humans by performing principal-component analysis (PCA) defined by a subset of Eurasian populations from the Human Origins dataset [8, 9] (Figure 1B). Our Romanian genomes were projected onto the first two PCA axes, together with a broad panel of ancient individuals (see Data S1 for sample details), augmented by two newly sequenced Spanish Mesolithic hunter-gatherer genomes (Chan_Meso, an Early Mesolithic dated at 9,131 ± 124 cal BP (calibrated age before present), and Canes1_Meso, a Late Mesolithic dated at 7,115 ± 130 cal BP) to provide better coverage for this important period (Table 1 and STAR Methods, Method Details). The three Romanian Mesolithic genomes clustered together with other Mesolithic samples, including the two new Spanish ones, and close to modern Northern European populations (Figure 1B). The Romanian Eneolithic genome GB1_Eneo, on the other hand, was placed in a different region of the plot, located in a unique position between European Mesolithic hunter-gatherers and Early Neolithic farmers on PC2. We confirmed the intermediate nature of this genome by estimating ancestral components using the clustering algorithm ADMIXTURE. Whereas the Romanian Mesolithic hunter-gatherers had a single major ancestral component shared with other WHGs, the Romanian Eneolithic sample was characterized by a mixture between this WHG component and a component maximized in Neolithic farmers (Figures 1C and S3B). Other admixed European Neolithic and Eneolithic/Chalcolithic farmers had at most 20%–30% WHG ancestry in ADMIXTURE analysis, and the Romanian Eneolithic is the only individual who is genetically predominantly Mesolithic (61.7%, 95% confidence interval [CI] 59.9%–63.4%) with a minority contribution from the Neolithic. We note that Gok2, a Swedish Neolithic sample, was originally estimated to have 77% hunter-gatherer ancestry [5], but in our analysis it has a much lower percentage (27.2%, 95% CI 25.1%–29.4%), in line with other recent analyses [23]. Although GB1_Eneo is chronologically close to the beginning of the Bronze Age, we did not find the green component (Figure 1C) characteristic of individuals from the Yamnaya culture, showing that the large hunter-gatherer component in this Eneolithic individual is unlikely to be due to admixture with incoming steppe pastoralists (Table 2).

**Table 2:** Key *D* Statistics of the Form *D*(A,B; X,Y)

A	B	X	Y	*D*	*Z* Score	Loci
**The Romanian Samples Form a Clade with Each Other (|Z| < 3; the Most Positive and Negative Statistics Are Shown for Each Comparison)**						

Mbuti	**Natufian**	**SC1_Meso**	**SC2_Meso**	0.03	1.635	26,749
Mbuti	**ElMiron**	**SC1_Meso**	**SC2_Meso**	−0.0281	−1.921	44,549
Mbuti	**GoyetQ116-1**	**SC1_Meso**	**OC1_Meso**	0.0272	1.896	41,197
Mbuti	**SC2_Meso**	**SC1_Meso**	**OC1_Meso**	−0.0283	−2.036	57,452
Mbuti	**Kostenki14**	**OC1_Meso**	**SC2_Meso**	0.023	1.964	79,553
Mbuti	**Armenia_EBA**	**OC1_Meso**	**SC2_Meso**	−0.0188	−2.192	77,923

**There Is Anatolian-Farmer-Related Admixture in the Romanian Eneolithic Sample as Compared to the Romanian Mesolithic Samples**						

Mbuti	**Anatolia_NW**	**SC1_Meso**	**GB1_Eneo**	0.0261	3.523	70,707
Mbuti	**Anatolia_NW**	**SC2_Meso**	**GB1_Eneo**	0.0273	4.469	101,212
Mbuti	**Anatolia_NW**	**OC1_Meso**	**GB1_Eneo**	0.0302	4.614	89,601

**The Romanian Eneolithic Sample Is Equally Related to Neolithic Farmers from Central Anatolia and Northwest Anatolia**						

Mbuti	**GB1_Eneo**	**Anatolia_NW**	**Anatolia_C**	−0.0038	−0.590	93,551

**The Spanish Mesolithic Sample Canes_Meso Forms a Clade with the Spanish Mesolithic Sample La Braña (|Z| < 3; the Most Positive and Negative Statistics Are Shown)**						

Mbuti	Belarusian	**La Braña**	**Canes1_Meso**	0.0206	2.81	73,417
Mbuti	**Armenia_EBA**	**OC1_Meso**	**SC2_Meso**	−0.0188	−2.192	77,923

**The Spanish Mesolithic Sample Chan Does Not Form a Clade with Other Spanish Mesolithic Samples; We Suggest that the Chan Lineage Did Not Directly Contribute Much to Later Populations (the Five Most Positive Statistics Using Modern Populations Are Shown)**						

Mbuti	Ukrainian	**Chan_Meso**	**La Braña**	0.0289	4.637	91,325
Mbuti	Croatian	**Chan_Meso**	**La Braña**	0.0286	4.53	91,325
Mbuti	Bulgarian	**Chan_Meso**	**La Braña**	0.0269	4.446	91,325
Mbuti	Russian	**Chan_Meso**	**La Braña**	0.0259	4.361	91,325
Mbuti	Greek	**Chan_Meso**	**La Braña**	0.0261	4.346	91,325
Mbuti	Czech	**Chan_Meso**	**Canes1_Meso**	0.036	5.185	83,744
Mbuti	Croatian	**Chan_Meso**	**Canes1_Meso**	0.0354	5.158	83,744
Mbuti	Belarusian	**Chan_Meso**	**Canes1_Meso**	0.0354	5.138	83,744
Mbuti	Lithuanian	**Chan_Meso**	**Canes1_Meso**	0.0363	5.064	83,744
Mbuti	Russian	**Chan_Meso**	**Canes1_Meso**	0.0326	4.965	83,744

**Our Romanian Mesolithic Samples Are Not Directly Representative of the HG Ancestry in the Eneolithic Sample GB1_Eneo**						

Mbuti	**GB1_Eneo**	**WHG**	**Romanian_HG**	−0.0179	−3.016	107,575

**The Romanian Hunter-Gatherer Samples Have Some Additional Eastern Hunter-Gatherer-Related Ancestry Relative to WHGs**						

Mbuti	**EHG**	**WHG**	**Romanian_HG**	0.0177	3.329	103,285

**We Do Not Detect Yamnaya Admixture in the Eneolithic Sample GB1_Eneo**						

Mbuti	**Yamnaya**	**Romanian_HG**	**GB1_Eneo**	−0.0118	−2.135	107,443
Mbuti	**Yamnaya**	**WHG**	**GB1_Eneo**	−0.0036	−0.728	108,273

Ancient samples are highlighted in bold. WHG includes the samples I0585, I1507, Loschbour, Bichon, Villabruna, Ranchot, Rochedane, and Canes1_Meso. EHG (Eastern hunter-gatherer) includes the samples I0124 and I0061. Other samples included in each ancient group can be found in Data S1.

**Figure 2:**
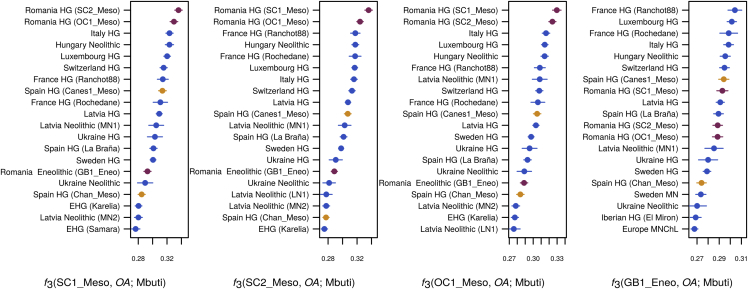
Outgroup *f*_3_ Statistics Outgroup *f*_3_ statistics of the form *f*_3_(*ancient1*, *ancient2*; Mbuti) for (A) SC1_Meso, (B) SC2_Meso, (C) OC1_Meso, and (D) GB1_Eneo. The highest 20 values of the test are given for each sample. The Romanian Mesolithic samples, SC1_Meso, SC2_Meso, and OC1_Meso, share the most drift with each other, followed closely by the WHG individuals. The Romanian Eneolithic sample, GB1_Eneo, also shares the most affinity with WHGs. See also Figure S3 and Data S2 for more values of this test.

We formalized these inferences by computing outgroup *f*_3_ in the form *f*_3_(ancient1, ancient2, Mbuti), thus estimating the amount of shared drift between pairs of ancient samples with respect to an African outgroup. Our three Romanian Mesolithic samples share the most drift with each other (Figure 2), followed by other WHGs, including our new Spanish samples. The genetic affinity among the Romanian Mesolithic samples was such that they form a clade to the exclusion of other ancient samples, as shown by *D* statistics of the form *D*(Romanian Mesolithic 1, Romanian Mesolithic 2, other_ancient, Mbuti) (Table 2). Interestingly, this was not the case for the Spanish Mesolithic samples, as Chan is somewhat divergent from Canes1 and La Braña (Figure S3A and Table 2), highlighting the genetic diversity of European Mesolithic hunter-gatherer lineages. The Romanian Eneolithic individual, on the other hand, once again showed a mix of affinities. Based on outgroup *f*_3_, the genomes that shared the most drift with this Eneolithic sample are WHGs, in line with the large amount of that ancestry detected in ADMIXTURE. However, its affinity to Neolithic samples is also relatively high compared to the Romanian Mesolithic samples (Data S2). This conclusion is supported by *D* statistics of the form *D*(GB1_Eneo, Romanian HG, Anatolian Neolithic, Mbuti), which indicate some Near Eastern ancestry (Table 2). Our three Romanian hunter-gatherer samples are not direct representatives of the hunter-gatherer component in GB1_Eneo (Table 2); however, this might be due simply to the geographic distance between the sites, especially given the observed heterogeneity among Spanish Mesolithic hunter-gatherers.

Analysis of runs of homozygosity (ROH) showed that the Eneolithic sample had a profile intermediate between Early Neolithic farmers and hunter-gatherers, consistent with the sample’s mixed origins (Figure S4A). Finally, we also tested the proportion of Neanderthal ancestry in each sample, which was consistent with the age of the sample (Figure S4B) [24].

### Stable-Isotope Analysis

In order to further assess the cultural affinities of these samples, we performed stable-isotope analysis on samples OC1_Meso, SC2_Meso, and GB1_Eneo, whereas isotope data from the other individuals involved in this study were collected from the literature [14, 15, 16]. The three Romanian Mesolithic individuals, OC1_Meso, SC1_Meso, and SC2_Meso, have isotopic values (Table 1 and Figures S4C and S4D) that indicate a high proportion of freshwater protein in an otherwise C_3_-based diet. By contrast, the lower δ^13^C and δ^15^N values of the Eneolithic individual GB1_Eneo suggest a mixed terrestrial/aquatic diet in which the aquatic component was lower than that consumed by the Iron Gates Mesolithic population. Although the GB1_Eneo results cannot be directly compared to the data from the Iron Gates, its stable-isotope values are similar to those found in some Neolithic individuals in the Iron Gates sites and at Vinča-Belo Brdo in Serbia, also on the Danube [11, 25, 26, 27].

## Discussion

The genetic analysis of the Eneolithic individual from Gura Baciului provides support for a scenario of complex interactions between hunter-gatherers and farmers in the Lower Danube Basin. At this stage, we cannot discern at what point during or after the Neolithic transition the observed hunter-farmer admixture occurred. Stable isotopic data from Lepenski Vir suggested early contacts between Neolithic immigrants and Mesolithic communities, whereby the first incomers changed the subsistence practices of the local fisher-hunter-gatherers, but not necessarily other cultural traits such as funerary practices [11, 28]. On the other hand, a resurgence of Mesolithic ancestry in the Late Neolithic has already been noted in other parts of Europe [6, 24], even though the relative contribution of this ancestry was limited compared to the levels we report for Romania. The fact that our Romanian hunter-gatherers are not direct representatives of the hunter-gatherer ancestry in GB1_Eneo (Table 2) provides some support for this latter scenario, but the geographic distance between the Mesolithic and Neolithic/Eneolithic sites might also explain this lack of a direct link. Although our results provide evidence for admixture between these communities, we do not know whether it resulted from the incoming farmers establishing their own communities into which the hunter-gatherers mixed or from small numbers of farmers joining existing hunter-gatherer communities.

Our analysis of uniparental markers provides a caveat on their use to infer the dynamics of interactions among different populations. Although two of our Romanian Mesolithic samples belong to mtDNA haplogroups (U5a and U5b), which are typical of that group, another Mesolithic individual of similar age and geographical origin (OC1_Meso) shares the same haplogroup (K1) with the much later Eneolithic individual GB1_Eneo and four previously described Middle/Late Neolithic and Eneolithic samples from Romania [29]. K1 has mostly been found in early farmers from Europe [6, 17, 18, 30, 31, 32] and has been hypothesized to have its origin in the Near East [1, 9, 18]. Although the latter hypothesis is not invalidated by our data, the occurrence of this haplogroup in a Mesolithic sample from Romania suggests that it entered the European gene pool clearly ahead of the Neolithic transition and should therefore not be used as a marker for tracking farmers of Anatolian origin. Similarly, all three Romanian Mesolithic individuals belonged to the Y chromosome haplogroup R1. This haplogroup is thought to have originated shortly after the Late Glacial Maximum (LGM) [33], but its frequency is found to increase greatly in Central Europe during the Late Neolithic and Bronze Age, leading to the hypothesis that these haplotypes expanded from the East only after ∼4.5 kya [6]. Its presence in all of our Romanian Mesolithic individuals and in older European human remains [7, 34] suggests that this haplotype was already found at high frequencies in certain regions of Europe well before then, even though this observation does not negate that the changes in frequency during the Bronze Age might reflect migration by steppe pastoralists.

The genome-wide data that we obtained from the Romanian Mesolithic and Eneolithic individuals suggest that the interaction between farmers and hunter-gatherers was much more complex than the simple picture of mostly demic replacement of hunter-gatherers by farmers that has been suggested by previous studies of ancient genomes from Western and Central Europe. The limited number of samples in this study prevents a formal investigation of the demographic processes that led to the observed patterns in *D* and *f* statistics. However, when our findings are considered together with the observation of an Early Neolithic individual from Hungary (KO1), whose ancestral component was completely derived from WHG [32], as well as the most recent finding that the Neolithic transition in the Baltic and Ukraine occurred with little or no input from farmers of Anatolian ancestry [7], a picture emerges in which the further north and east we go from the Carpathian Basin, the lesser the role of a demic diffusion in the spread of the Neolithic traits, thus implying an increase in importance of cultural exchange. Interestingly, a similar gradient has been suggested for East Asia, with high levels of genetic continuity with hunter-gatherer populations in the northern parts of the region and admixture between this lineage and incoming farmers further south [35]. We do not know what determined this cline. We could speculate, though, that the Neolithic package, which was developed in the Near East, might have been somewhat unsuitable for the new climates and environments encountered in the northeast of Europe, leading to a progressive mixing with local hunter-gatherers and acquisition of local knowledge, with the eventual breakdown of demic diffusion and the spread of isolated Neolithic traits by cultural diffusion. Another scenario is that hunter-gatherers were in higher densities in these eastern regions and hence that interactions between hunter-gatherers and farmers were more frequent than in regions further west.

## STAR★Methods

### Key Resources Table

**Table:** 

REAGENT or RESOURCE	SOURCE	IDENTIFIER
**Biological Samples**		

SC1_Meso	“Vasile Pârvan” Institute of Archaeology, Romanian Academy, 11 Henri Coandă St	M95/2
SC2_Meso	“Vasile Pârvan” Institute of Archaeology, Romanian Academy, 11 Henri Coandă St	M96/3
OC1_Meso	“Olga Necrasov” Centre for Anthropological Research of the Romanian Academy, Iaşi Branch	M24
GB1_Eneo	“Olga Necrasov” Centre for Anthropological Research of the Romanian Academy, Iaşi Branch	M1
Chan_Meso	University of A Coruna, Instituto Universitario de Xeoloxia Isidro Parga Pondal (IUX)	Elba
Canes1_Meso	Universidad Complutense de Madrid. Department of Zoology and Physical Anthropology. Museo Arqueológico de Asturias. Oviedo, Spain	I-A
Canes2_Meso	Universidad Complutense de Madrid. Department of Zoology and Physical Anthropology. Museo Arqueológico de Asturias. Oviedo, Spain	II-A

**Chemicals, Peptides, and Recombinant Proteins**		

Guanidinium hydrochloride 99%, M 95,53 g/mo	Roth	Cas#50-01-1
Guanidinium thiocyanate (GuSCN) 99%, M 118,16 g/mol	Roth	Cas#593-84-0
Sodium acetate	Sigma Aldrich	Cas#127-09-3
Silicon dioxide 500 G	SLS	Cas#7631-86-9
Isopropanol	Sigma-Aldrich	Cas#67-63-0
Tween-20	Sigma-Aldrich	Cas#9005-64-5

**Critical Commercial Assays**		

High sensitivity DNA chip (Bioanalyzer 2100)	Agilent	Cat#5067-4626

**Deposited Data**		

SC1	This paper	ENA: PRJEB20616
SC2	This paper	ENA: PRJEB20614
OC1	This paper	ENA: PRJEB20614
GB1	This paper	ENA: PRJEB20614
Chan	This paper	ENA: PRJEB20614

**Oligonucleotides**		

IS1_adapter.P5: 5′-A^∗^C^∗^A^∗^C^∗^TCTTTCCCTACACGACGCTCTTCCG^∗^A^∗^T^∗^C^∗^T-3′ (here and below, ^∗^ indicates a PTO bond)	[36]	Sigma Aldrich
IS2_adapter.P7: 5′-G^∗^T^∗^G^∗^A^∗^CTGGAGTTCAGACGTGTGCTCTTCCG^∗^A^∗^T^∗^C^∗^T-3′	[36]	Sigma Aldrich
IS3_adapter.P5+P7: 5′-A^∗^G^∗^A^∗^T^∗^CGGAA^∗^G^∗^A^∗^G^∗^C-3′	[36]	Sigma Aldrich
IS4: 5′-AATGATACGGCGACCACCGAGATCTACACTCTTTCCCTACACGACGCTCTT-3′	[36]	Sigma Aldrich
P7 indexing: 5′-CAAGCAGAAGACGGCATACGAGATxxxxxxxxGTGACTGGAGTTCAGACGTGT-3′	[36]	Sigma Aldrich

**Software and Algorithms**		

Illumina Pipeline v1.4	Illumina	https://support.illumina.com/downloads.html
SeqPrep	John St. John	https://github.com/jstjohn/SeqPrep
Burrows-Wheeler Aligner (BWA) 0.7.5a-r405	[37]	http://bio-bwa.sourceforge.net/
Picards-tools-1.98	Broad Institute	https://sourceforge.net/projects/picard/files/picard-tools/1.98/
GATK-3.0-0	[38]	https://software.broadinstitute.org/gatk/download/archive
Samtools-0.1.19	[39]	https://sourceforge.net/projects/samtools/files/samtools/0.1.19/
mapDamage	[40]	https://ginolhac.github.io/mapDamage/
Haplogrep	[41]	http://haplogrep.uibk.ac.at/
ANGSD	[42]	http://popgen.dk/angsd/index.php/Contamination
Yfitter	[43]	https://sourceforge.net/projects/yfitter/
EIGENSOFT 5.0.1	[44]	https://github.com/DReichLab/EIG
ADMIXTOOLS	[44]	https://github.com/DReichLab/AdmixTools
ADMIXTURE	[45]	https://www.genetics.ucla.edu/software/admixture/
PLINK	[46]	https://www.cog-genomics.org/plink2

**Other**		

Proteinase K	Promega	Cat#MC5005
MinElute PCR Purification Kit	QIAGEN	Cat#28004
Mobicol M1050	MoBiTec GmbH	Cat#S10011
Filter (small) 10 μm pore size	MoBiTec GmbH	Cat#M2110
Zymo-spin V column extension reservoir	Zymo Research	Cat#C1016-25
T4 polymerase	Fermentas/ThermoFisher	Cat#EP0062
T4 Polynucleotide kinase	Fermentas/ThermoFisher	Cat#EK0031
Buffer Tango	Fermentas/ThermoFisher	Cat#BY5
ATP	ThermoFisher	Cat#R0441
PEG-4000	Sigma Aldrich	Cat#1546569
T4-ligase	Fermentas/ThermoFisher	Cat#EL0011
Bst-polymerase, large fragment (supplied with 10X ThermoPol reaction buffer)	New England BioLabs	Cat#M0275 S
Accuprime SuperMix I	ThermoFisher Scientific	Cat#12342010

### Contact for Reagent and Resource Sharing

Further information and requests for reagents may be directed to, and will be fulfilled by the Lead Contact, Michael Hofreiter (michi@palaeo.eu).

### Method Details

#### Description of the archaeological context: sites and samples

The sampling was agreed in order to minimize damage to the archaeological remains and all samples were collected and analyzed in accordance with the research ethics policies and procedures of the academic institutions involved in the research.

##### 1. Schela Cladovei

Schela Cladovei (Romania), is a large, open-air site on an Early Holocene terrace adjacent to the River Danube (44.6258333, +22.6066666), c. 7 km downriver from the Iron Gates I dam. Discovered in 1964, the first excavations were undertaken by the Romanian archaeologist Vasile Boroneanţ. From 1992 onward, the excavation became a joint Romanian–British research project, co-directed by V. Boroneanţ, A. Boroneant and C. Bonsall.

Archaeological remains in the areas investigated relate mainly to the Late Mesolithic and Early Neolithic, with sporadic evidence of later (Iron Age and Medieval) occupation. A large series of single-entity AMS ^14^C dates on animal and human remains [10] places the Late Mesolithic occupation between c. 9,150 and 8,250 cal yBP, and the Early Neolithic occupation between 7,950 and 7,550 cal yBP.

At least 75 burials, containing the remains of over a hundred individuals, have been excavated from the Schela Cladovei site so far, most of them dated to the Late Mesolithic. The two individuals from Schela Cladovei included in this study were from burials M95/2 (Laboratory ID: SC1_Meso) and M96/3 (Laboratory ID: SC2_Meso), both found among 21 burials uncovered in an area c. 25 m x 4 m immediately adjacent to the Danube riverbank between 1991 and 1996. Of those 21 burials, which included adults and children, 11 (all adults) have single-entity AMS ^14^C dates from the Oxford Radiocarbon Accelerator Unit (ORAU). The dating was done prior to the use of ultrafiltration by ORAU. The calibrated ages (after correction for the Danube Freshwater Reservoir Effect, FRE) range between 8,950 and 8,550 cal yBP.

Burials M95/2 and M96/3 are both extended inhumation burials. Body position, stratigraphic relationship to Early Neolithic pit features, and δ^15^N values of > 14‰ all point to a Late Mesolithic age, which is confirmed by the ^14^C date (OxA-8583) for SC1_Meso.

1. SC1_Meso: Adult male, age-at-death 35-45 (dental attrition). The skeleton was lying on the right side, with the legs slightly flexed. The burial was truncated by an Early Neolithic pit, which removed the mid-section of the skeleton. The distal ends of both femurs and the lower legs were missing, possibly removed by another pit feature. The skeletal remains were dated to 8,380 ± 80 yBP (OxA-8583) and corrected to 7,960 ± 96 yBP (9,075-8,553 cal yBP) after considering the FRE [47] (Table S1). The FRE is related to fish consumption; since fish from the Danube are relatively depleted in ^14^C, radiocarbon dates for fish bones and the bones of animals (including humans) that consumed fish are older than their archaeological context. This age offset can be quantified and corrected for based on the δ^15^N ratio, as described by [47].
2. SC2_Meso: Child, 5-7 years of age at death. There is no ^14^C date for SC2_Meso, but it belongs to the same (Late Mesolithic) burial cluster as SC1_Meso and all dates for those burials are statistically indistinguishable at the 2-sigma level [10, 48]; thus SC2_Meso can be expected to date to the same time period as SC1_Meso.

##### 2. Ostrovul Corbului

The Ostrovul Corbului site is also situated in the Iron Gates region of southwestern Romania (44.5154854, +23.52087725) on a former island in the Danube River, 28km downstream of Schela Cladovei. Settlement remains, individual graves and a cemetery belonging to various prehistoric periods (Mesolithic, Neolithic, Eneolithic, Bronze Age, and Iron Age) were identified during several excavation campaigns between 1973 and 1984 [49]. Seven inhumation burials (no. 2, 9, 18, 24, 25, 30 and 32) were found in an area with Mesolithic and Early Neolithic settlement remains at the SW (downstream) end of the island. These were previously interpreted as Early Neolithic in date, but AMS ^14^C dating has shown that burials no. 2, 25 and 30 belong to the Middle Mesolithic between about 9.7–9.3 kya, while burial no. 32 dates to the Late Mesolithic around 8.6 kya [10].

The individual from Ostrovul Corbului included in this study (laboratory ID: OC1_Meso) comes from burial no. 24. Only the upper part of the skeleton was preserved, with the bones in anatomical position. The lower part of the skeleton appears to have been destroyed by a later pit feature. From the surviving portion of the skeleton, burial 24 was interpreted as an extended, supine inhumation oriented with the head toward the east [49]. The skeleton is that of an adult male, with age at death estimated at 30-35 year and stature at 172 cm [50, 51]. The AMS ^14^C date obtained for this study was 8,277 ± 34 yBP (MAMS-28615). After applying a FRE correction using Method 1 of [47], this converts to a 2-sigma calibrated age range of 8,972–8,435 cal BP (Table S1), which overlaps with the calibrated age of SC1 from Schela Cladovei.

##### 3. Gura Baciului

The Gura Baciului site (46.7877247, +23.5208770) is located on a terrace of the Suceag creek, in Transylvania, near Cluj Napoca city (Cluj county). Excavations by N. Vlassa (in 1960, 1962, 1965, 1967-1971) and Gh. Lazarovici (in 1990–1993) uncovered the remains of a Starčevo-Criş settlement with huts or houses, pits and concentrations of domestic refuse [52]. Food remains recovered in the excavations indicate a typical Early Neolithic farming economy based on crop (cereals, etc.) and livestock (cattle, sheep/goat and pig) husbandry. The material culture remains included pottery, lithic artifacts (e.g., flint, obsidian, ground stone axes, seed grinding equipment), anthropomorphic and zoomorphic figurines, and various kinds of personal ornaments (clay bracelets and buttons, bone rings, *Spondylus* shell bracelets and pendants). Based on analysis of the pottery, the site was considered to have been occupied more-or-less continuously throughout the greater part (stages IB–IVB) of the Starčevo culture time range [51]. There are very few ^14^C dates on finds from the Gura Baciului excavations, but ^14^C results from other Starčevo culture sites in Transylvania indicate a time range for phases I–IV of c. 7950–7350 cal BP (6,000–5,400 cal BCE) [53].

Seven primary inhumation burials (no. 1, 2, 3, 4, 5, 6, and 9), a cremation grave (no. 7) and a secondary burial (no. 10) were found within the area of the Starčevo culture settlement, while ‘loose’ human bones (a complete skull [M8] or skull fragments) were discovered in domestic contexts (e.g., pits and houses) belonging to the ‘cultural layer’. Most of the primary inhumations were buried in crouched positions, on the left or right sides, with varying orientations. These crouched inhumations were assumed to be contemporaneous with the Starčevo culture occupation, although few if any chronologically diagnostic items (‘burial offerings’) were recovered from the graves. Moreover, across Southeast Europe, Neolithic and later prehistoric people tended to bury their dead in formal disposal areas on the periphery of (or some distance away from) their living areas, and it was not unusual for graves to be dug into abandoned living areas.

Skeletal remains from grave no. 6 were dated to 6,400 ± 90 yBP (7,495–7,159 cal yBP; Lv-2157; [52, 54]). Recently, two other graves from Gura Baciului were dated: grave no. 2 – 6,350 ± 40 yBP (7,415–7,174 cal yBP) and grave no. 3 – 6,370 ± 40 yBP (7,420–7,182 cal yBP; [29]). However, there are no associated carbon and nitrogen stable isotope values from which to assess the individuals’ diet. Assuming no freshwater reservoir effect, this date places the burial around the end of the Starčevo culture period and it is distinctly possible that the burial was emplaced after the living area had been abandoned. The only ^14^C date from Gura Baciului from a *non-burial* context is an AMS measurement on animal bone from a pit feature (possibly a dwelling structure) of 7,140 ± 45 yBP (8,035–7,860 cal BP; GrA-24137), which predates burial no. 6 by c. 600 years.

The individual from Gura Baciului included in this study (laboratory ID: GB1_Eneo) comes from grave no. 1 (archaeological ID: M1). This grave was a chance find when the section of an older trench collapsed. Discovered in 1962 near a pit-house, it is a primary inhumation of a single individual (GB1_Eneo) in anatomical connection, deposited in the crouched position on the left side, with ESE–WNW orientation (head to east). The reported grave goods comprise ten flint flakes in the feet area and a bone awl and two ochre fragments in the cheek and hip areas. Eighty-three fragments of animal bones (of *Bos taurus, Ovis aries/Capra hircus*, *Cervus elaphus, Bos primigenius*), mollusca, broken stones and numerous ceramic fragments supposedly formed a “bed” underneath the body [55, 56, 57, 58]. Among this material were three “loose” human bones of an 11–13 year old child [56]. The skeleton from grave no. 1 is that of an adult female, around 155 cm tall [50, 56]. The AMS ^14^C date of 4,621 ± 28 yBP (5,456–5,299 cal BP, MAMS-28614, this study) (Table S1) places this burial in the Eneolithic period, and is further evidence of the post-abandonment use of the Starčevo culture settlement as a formal burial area. The δ^15^N value of +12.7‰ implies the individual had a high trophic level diet that may have included significant amounts of fish, which in turn would likely result in a ^14^C age that is older than the archaeological context (i.e., that includes a reservoir offset). Thus, the ^14^C date should be regarded as a maximum age for this individual. The Eneolithic period date for burial no. 1 is not entirely surprising, since in early survey work in the Gura Baciului locality (prior to 1942 — items from a private collection donated to the museum in 1942), traces of Eneolithic and EBA activity were recorded [52].

##### 4. Chan do Lindeiro

The Chan do Lindeiro karstic system (42.7343714,-7.0305368, Pedrafita, Lugo, Spain) is a large vertical fracture associated with a small doline. The human remains from this site belong to a single individual (laboratory ID: Chan_Meso; archaeological ID: Elba) and were found with no funerary context in a deep cave gallery associated with a collapsed sinkhole [58]. The skeletal fragments were scattered among the collapse debris and completely disarticulated. Only a few bones were recovered: a partial neurocranium, a single tooth, some vertebrae, both clavicles, fragments of ribs, a partial ulna, both femora, a partial tibia and one metatarsal.

No other human remains or goods or lithic tools were found. The only other findings at the site are the remains of three small-sized aurochs. The sedimentological analysis suggests that the collapse occurred in a single episode, and the radiocarbon ages for the human and the aurochs overlap [16], suggesting that all these remains might be somehow related.

Chan_Meso (archeological ID: Elba) is a female individual of small size (around 153 cm tall and 56 kg in weight, according to [16]) but shows marked muscle scars and several pathologies indicating hard physical work from youth [59]. The only tooth recovered shows a large caries lesion. Independent ^14^C dates are available from the skull (7,995 ± 70 ^14^C years yBP; Ua-13398; [60]) and the tibia (8,236 ± 51 ^14^C yBP, Ua-38115; [16]). The weighted mean of the two ^14^C ages is 8,155 ± 42 yBP (R-combine, Oxcal 4.2) and the 2-sigma calibrated age range calculated using OxCal 4.2 and the IntCal13 dataset is 9,255–9,007 cal yBP (Table S1).

##### 5. Los Canes

Los Canes cave is located on the southern slope of Sierra de Cuera, a mountain chain in the eastern part of the Asturias region in northern Spain (43.3550277, −4.7166763). There is evidence of human occupation from the Solutrean until the Bronze Age, and several human remains have been found associated with these periods [61, 62, 63, 64].

Three well preserved burials belonging to the Mesolithic period were found at the entrance of the cave:

Burial I: This corresponds to a woman of advanced age (laboratory ID: Canes1_Meso; archaeological ID: I-A) that was AMS ^14^C dated to 6,265 ± 75 yBP (AA-5294; [62]) and 6,160 ± 55 yBP (OxA-7148; [65]). The weighted mean of the two measurements is 6,197 ± 45 yBP (R-combine, Oxcal 4.2), giving a 2-sigma calibrated age calculated using OxCal 4.2 and the IntCal13 dataset of 7,245–6,985 cal yBP. Some objects have been interpreted as grave goods: a red deer (*Cervus elaphus*) scapula, an ungulate rib and three perforated red deer canines. Also, a large quantity of shells of *Cepaea nemoralis* was found all around the skeleton, probably an intentional deposit [61, 62].
Burial II: Remains of two individuals interred at different times were found in this grave [61, 62]. Most of the skeleton of the earlier one (individual II-B, an adult), dated to 6,860 ± 65 yBP (AA-5295 [62]; 7,826–7,583 cal yBP) was probably removed when the later individual (II-A) was buried, and only the feet in anatomical connection and some scattered bones and teeth were preserved. The skeleton of individual II-A, a subadult male (laboratory ID: Canes2; archaeological ID: II-A), was found in anatomical connection, lying on his left side in flexed position. It was dated by AMS ^14^C to 6,770 ± 65 yBP (AA-5296; [62]), 7,025 ± 80 yBP (AA-11744; [62]) and 7,208 ± 38 yBP (OxA-23185; [63]). The weighted mean of these three measurements is 7,092 ± 31 yBP (R-combine, Oxcal 4.2) and the 2-sigma calibrated age range is 7,974–7,850 cal yBP. Several grave goods were associated with the II-A skeleton: two frontlets of female ibex (*Capra pyrenaica*), a long cobble with traces of red colorant, a long, pointed bone, a perforated antler, and a pecked cobble, possibly representing a human head. Pendants (most of them made of *Trivia* sp. shells) were found around the head and shoulders of this individual, suggesting that they were sewn to a dress [66].
Burial III: This grave contained the remains of a complete adult male (III-A), dated to 6,930 ± 95 yBP (AA-6071 [62]; 7,944–7,609 cal yBP). Earlier skeletal remains from an infant (III-B) associated with coeval bones of the Iberian chamois (*Rupicapra pyrenaica*), red deer (*Cervus elaphus*) and wild boar (*Sus scrofa*) were found over III-A’s knees, indicating the removal of a previous burial [63, 67].

The paleoanthropological characteristics of the Los Canes fossils place them within the variability described for the last hunter-gatherers of the Late Upper Paleolithic/Early Mesolithic of Western Europe [67]. All adult individuals from Los Canes showed marked muscle insertions, and especially I-A and III-A individuals had oral pathologies including caries, ante-mortem tooth loss and dental calculus; marked wear of the crown surfaces was also observed [64]. Because of the availability of petrous bones, individuals I-A and II-A were selected for genetic analysis. After estimating the percentage of endogenous DNA, only I-A was selected for genome sequencing.

#### Sample preparation, DNA extraction and library building

All pre-amplification DNA procedures were carried out in dedicated aDNA laboratories at the University of York (UK). All laboratory tools used to process the samples were either sterile and disposable or decontaminated with bleach (concentration 1:5) and exposed to UV light for 24h before being used. DNA was extracted from the walls forming the channels of the inner ear within the petrous bone, which tend to preserve comparatively high percentages of endogenous DNA [32, 68]. In total, 7 petrous bones were processed at this step: 6 from human remains associated with Mesolithic sites in Romania (SC1_Meso, SC2_Meso and OC1_Meso) and Spain (Chan_Meso, Canes1_Meso and Canes2_Meso); and 1 from a Neolithic/Eneolithic site in Romania (GB1_Eneo). Before extraction, the fragments of petrous bones were treated for decontamination: they were first exposed to UV light for 10 min on each side, followed by physical removal of the surface with a Dremel drill, and finally, once the surfaces were clean, they were exposed again to UV light for another 10 min.

The bone fragments were ground to powder using a mortar and pestle and one DNA extract was obtained from each sample. Because the samples from Romania and Spain were sampled and extracted at different times, we followed two different protocols ([69] and [70], respectively). From the Romanian samples, DNA was extracted following [69], starting from approximately 250 mg of bone powder. Each sample was incubated in rotation with 5 ml of extraction buffer, consisting of 0.45 M EDTA (pH 8.0) and 0.25 mg/ml of proteinase K. After overnight incubation, the samples were centrifuged and the supernatant was transferred to a new tube with 2.5 ml of binding buffer (5 M guanidinium thiocyanate (GuSCN) and 300 mM sodium acetate) and 100 μl of silica suspension. The mix was incubated for 3 hr in gentle rotation at room temperature. After incubation, the tubes were centrifuged, the supernatant discarded and the silica pellet was resuspended in 400 μl of binding buffer and transferred into a clean Mobicol column with an extra 10 μm filter (MoBiTec GmbH). The columns were centrifuged and then washed twice with 450 μl of washing buffer (50% ethanol, 125 mM NaCl, 1 × TE) in order to remove salts and PCR-inhibitors. Finally, the silica pellet was dried and the DNA eluted into a clean tube using 50 μl of TE.

The Spanish samples were extracted following a more recent protocol from [70], which optimizes the recovery of short DNA fragments from small quantities of bone powder. From each sample, 50 mg of bone powder were digested in 1 ml of an extraction buffer consisting of 0.45 M EDTA (pH 8.0) and 0.25 mg/ml of proteinase K. After overnight incubation, 1 ml of supernatant was added to 13 ml of binding buffer (5M Guanidine hydrochloride (MW 95.53), 40% Isopropanol, 0.05% Tween-20, 9 mM Sodium Acetate) and poured into a binding apparatus consisting of an extension reservoir (Zymo Research) fitted to a MinElute silica spin column (QIAGEN). The binding apparatus was placed into a 50-ml falcon tube and centrifuged. During the centrifugation, the silica based membrane in the MinElute column will retain the DNA molecules. This filter is washed by adding 650 μl of PE buffer (QIAGEN). Finally, the filters are dried by centrifugation and the DNA molecules are eluted by adding a total of 25 μl of TET buffer (1 M Tris-HCL, pH 8.0, 0.5 M EDTA, 10% Tween-20).

Both extraction experiments were performed including two blank controls each.

Illumina libraries were built following the protocol described by [36], with the modifications suggested by [71], which minimize the number of purification steps by introducing heat inactivation of the enzymes between consecutive reactions. We used 20 μl of DNA extract as template to build one sequencing library per sample. As first step, we performed the blunt end repair of overhang strands with enzymes T4 Polynucleotide kinase and T4 polymerase in the following reaction mix: 1x Buffer Tango, dNTP (100 μM each), 1 mM ATP, 0.1 U/μl T4 PNK, 0.1 U/μl T4 Polymerase. After 20 min of incubation at 25°C, the mix was heat up to 72°C for 20 min, in order to inactivate the enzymes. Following, the adaptor mix (1x T4 ligation buffer, 5% PEG-4000 and a mix 0.5 μM of the paired end adapters P5 and P7) was added to the former reaction, without any purification step through columns. Just before starting the incubation, 1.25 μl of T4 ligase (final concentration 5 U/μl) were added to each tube to complete the adaptor mix reaction. The tubes were incubated for 30 min at 22°C and then the volume was filtered through silica-based MinElute columns (QIAGEN). In the next step, the Bst polymerase was used to fill the nicks and to complete the double strand sequence of the adapters (reaction mix: 1x Thermopol buffer, dNTP (250 μM) and 0.3 U/μl Bst polymerase). The mix was incubated at 37°C for 20 min and then heated up to 80°C for 20 min.

After the incubation, each library was indexed and amplified in three parallel reactions, without any filtration step between the adaptor fill-in and the amplification. We used 5 μl of library as template for each reaction and the primers IS4 (5′-AATGATACGGCGACCACCGAGATCTACACTCTTTCCCTACACGACGCTCTT-3′) and P7 indexing (5′-CAAGCAGAAGACGGCATACGAGATxxxxxxxxGTGACTGGAGTTCAGACGTGT-3′). The P7 indexing primer includes a barcode sequence of 8 nucleotides (denoted by the eight x). We used a different barcode for each sample, so the libraries could be pooled and sequenced together. Library amplifications were carried out using Accuprime Supermix I (ThermoFisher Scientific), which includes a polymerase that is able to read over uracils. The PCR mix was prepared for a total volume of 25 μl following the instructions from the manufacturer. Amplification conditions were as follows: 95°C for 5 min; 12 cycles of: 95°C for 15 s, 60°C for 30 s, 68°C for 30 s and, finally, an extension of 68°C for 5 min. The three PCR reactions from each sample were pooled and purified on a MinElute column (QIAGEN), with a final elution in 15 μl of EB buffer. The amplified libraries were visualized on agarose gels and quantified on a Bioanalyzer 2100 with the High Sentitivity DNA chip (Agilent).

The endogenous DNA content of the libraries was tested by sequencing on an Illumina MiSeq platform at TrinSeq (Trinity Genome Sequencing Laboratory, Trinity College Dublin, Ireland), using 50 bp single-end sequencing. Only samples with percentages of endogenous DNA higher than 20% were selected for further sequencing (Table S1): SC1_Meso, SC2_Meso, OC1_Meso, Chan_Meso, Canes1_Meso and GB1_Eneo.

#### Stable isotope analysis

Stable isotope data were available from the literature for Chan_Meso, Canes1_Meso and SC21_Meso. For individuals OC1_Meso, SC2_Meso and GB1_Eneo, we sampled approximately 0.5 g of bone and extracted collagen following [72]. The ‘collagen’ was then lyophilized before weighing for isotopic analysis. Each sample was analyzed in triplicate using a Costech elemental analyzer coupled in continuous-flow mode to a Finnigan isotope ratio mass spectrometer (Delta V). Carbon and nitrogen isotopic ratios were measured on the delta scale in comparison to international standards, VPDB and AIR respectively, in units of ‘permil’ [73, 74]. Repeated-measurements on international and in-house standards showed that the analytical error was < 0.2‰ for both carbon and nitrogen.

All samples contained collagen deemed to be of good quality as it fulfilled the following criteria: an atomic C:N ratio of 2.9 to 3.6 [75]; a ‘collagen’ yield of >1% by mass; final carbon yields of >13%; and final nitrogen yields of >4.8% [76]. The results from these isotope analyses are described in the main text and illustrated in Figures S4C and S4D. Figure S4C shows a bivariate plot of δ13C and δ15N values for the studied Romanian humans compared against the overall ranges for Late Mesolithic and Early Neolithic humans and fauna (terrestrial ungulates and fish) from the Iron Gates. The Romanian Mesolithic individuals have high δ13C and δ15N values indicating a high proportion of freshwater protein in an otherwise C3-based diet. By contrast, the Eneolithic individual GB1_Eneo had values similar to those found in Neolithic individuals from the Iron Gates sites, reflecting a more terrestrial C3-based diet but still with an important aquatic component [11, 25, 26, 77].

Isotopic values from the individual Chan_Meso have been published [16]: δ13C = −20.5‰ and δ15N = 8.4‰. These values are relatively low for a hunter-gatherer, lower than those of the La Braña individuals (δ13C = −19.3 and −18.9‰, δ15N = 10.6 and 10.4‰; [78]), and more similar to Canes1_Meso (δ13C = −20.0‰ and δ15N = 7.9‰; [15]) and later Neolithic farmers from Portugal [79]. Moreover, the stable isotope signatures of the Chan_Meso and Canes1_Meso individuals differ considerably from other Mesolithic hunter-gatherers from the Cantabrian area [15], with the La Braña individuals situated in an intermediate position (Figure S4D). However, the Cantabrian Mesolithic individuals came from coastal sites associated with shell middens, like the Portuguese Mesolithic sites studied in [79], while Canes1_Meso, Chan_Meso and La Braña are relatively far from the coast, in inland mountainous areas. Judging by the isotopic signatures, their economic strategies did not include marine diet inputs, at least for Canes1_Meso and Chan_Meso.

Isotopic values cannot be compared directly, as they depend largely on local values in soils, which in turn depend on altitude, insulation, rainfall and other environmental parameters [80]. For a better interpretation, it is necessary to establish a local isotopic baseline, ideally using contemporaneous faunal values [81]. When comparing the human values with those of contemporaneous red deer from El Mirón, a nearby site in the Cantabrian area [77] and the three aurochs in Chan do Lindeiro cave, the isotopic offset between ungulates and both humans is equivalent to a complete trophic level, confirming a diet based largely on terrestrial ungulates but with an appreciable contribution from C3 vegetables (Figure S4D).

### Quantification and Statistical Analysis

#### Processing and alignment of NGS data

Six libraries were selected for HiSeq sequencing after the first screening: SC1_Meso, SC2_Meso, OC1_Meso and GB1_Eneo from Romania and Chan_Meso and Canes1_Meso from Spain. Each library was sequenced on one lane of an Illumina HiSeq2000 platform at Beijing Genomics Institute (BGI), except Chan_Meso, which was sequenced on two lanes. Sequencing was performed with 100 cycles in paired end mode.

BCL files were converted to fastq format using the Illumina base-calling pipeline (Illumina Pipeline v1.4). Raw reads were assigned to the corresponding samples based on the index sequence included in the adaptor P7, allowing no mismatches in the first base of the sequence and a single mismatch at any other position [82, 83]. The software SeqPrep (https://github.com/jstjohn/SeqPrep) was used to trim the adapters and merge the forward (R1) and reverse (R2) reads. Default parameters were used for minimum base pair overlap to merge the reads (-o 15) and the quality score cutoff for mismatches to be counted in an overlap (-q 13), while a minimum length of 30 was set as threshold to output a merged read.

The merged reads that passed all quality filters were mapped to the human reference genome using the software Burrows-Wheeler Aligner (BWA) version 0.7.5a-r405 [37], with default parameters and seed option disabled (-l 1000). We used hg19 (GRCh37 build) as reference genome, but the mitochondrial reference was replaced with the revised Cambridge Reference Sequence (rCRS, NC_012920; [84]).

Clonal sequences were removed using MarkDuplicates.jar from picards-tools-1.98 (http://broadinstitute.github.io/picard/) and indels were realigned using the tools RealignerTargetCreator and IndelRealigner from GATK-3.0-0 [38]. The resulting bam files were filtered for a minimum mapping quality of 30 using Samtools −0.1.19 [39]. Finally, the tool GenomeAnalysisTK.jar from GATK-3.0-0 was used to calculate the depth of coverage of the bam files. Table S2 summarizes the NGS output for each of the samples.

#### Test of DNA authenticity

Negative controls were included during the wet lab stages and sequenced together with the samples in the MiSeq test run (Table S1). A total of four blanks were sequenced for each protocol: eBL is the pool of the blanks 1 and 2 of the DNA extraction, and liBL is the pool of the blanks 1 and 2 from the process of building these extracts into libraries. The suffix *r* indicates blanks from samples extracted following [69], while *d* denotes [70]. The percentage of reads in the blanks aligning to the human reference sequence ranged from 0.25 to 0.9% (Table S1). Furthermore, for each sample, we analyzed patterns of molecular damage and presence of contaminants in monoparental chromosomes as explained below.

##### Read length and molecular damage

Because of molecular damage, ancient DNA molecules have short read length distributions and specific nucleotide misincorporations [85, 86, 87]; these two criteria can be used to verify the antiquity of DNA molecules. Patterns of molecular damage were estimated using mapDamage [40, 88]. Figure S1A illustrates the mapDamage output with the percentages of C to T and G to A nucleotide misincorporation rates at 5′ and 3′ ends, respectively, for each of the six samples, which ranged between 14% and 28%. We estimated the distribution of read lengths before (using a custom awk script on the fastq files) and after mapping (using the ReadLengthDistribution tool from GATK), Figure S1B. The average read length was between 79 and 102 for unmapped reads and between 62 and 109 for mapped reads. Reads from Chan_Meso and Canes1_Meso are shorter than those from the Romanian samples, probably because the latter were obtained with the [70] protocol, which is specifically designed for the recovery of short fragments.

##### Mitochondrial DNA authentication

Possible contamination from modern human DNA was assessed by investigating the frequency of non-consensus calls in the mitochondrial DNA (mtDNA); specifically, we focused on haplogroup defining positions recovered from Haplogrep (http://haplogrep.uibk.ac.at/), as these are known to be polymorphic in humans (monomorphic positions are less informative, as a non-consensus call would be more likely to be due to damage or miscalling). For each sample, samtools −0.1.19 [39] view option was used to extract the reads mapped to the rCRS to a single bam file, from which we called polymorphic positions using samtools mpileup tool. We computed two estimates for each sample: the percentage of non-consensus calls, which includes both contamination and potential molecular damage, %(C+MD), and the percentage excluding potentially damaged bases, %C, excluding transitions C to T and G to A (Table S2).

##### X chromosome based authentication

The X chromosome is a uniparental chromosome in males, providing the opportunity to quantify contamination following the same logic used for mtDNA. We used ANGSD [42], which implements the method described in [89], to assess the level of X chromosome contamination in our three male samples (SC1_Meso, SC2_Meso and OC1_Meso). Parameters recommended on the ANGSD website (http://popgen.dk/angsd/index.php/Contamination) were used with the minimum base quality threshold set to 30. Table S2 reports contamination estimates based on two tests: test 1, which uses all high quality reads per sample and then evaluates the rate of contamination based on a maximum likelihood approach; and test 2, which samples a single read per site to calculate the contamination rate. Low contamination ratios were found with both methods, with percentages around 1% for SC1_Meso and SC2_Meso and 2% for OC1_Meso (Table S2).

#### Molecular sex determination

Sex was determined by evaluating the ratio (Ry) of reads aligning to the Y chromosome (nY) compared to the total number of reads aligning to the sex chromosomes (nX + nY), i.e., Ry = (nY/nY+nX), as described in [90]. We observed that the highest fraction of reads aligning to the Y chromosome were Ry = 0.0046 in females and Ry = 0.098 in males (Table S2). All samples could be assigned to one of the two sexes with confidence higher than 95%, and all the genetic assignments were consistent with morphological identifications.

#### Analysis of phenotypic traits

We used mapDamage to rescale the base quality score of T and A according to their probability of resulting from molecular damage (C to U deaminations). We used these rescaled bam files and Samtools mpileup to call variants at specific positions that have been identified as having a role in determining phenotypic traits. For SNP calling, we only used bases with quality ≥30 and positions covered by at least 3 reads. Owing to the low coverage of some of the genomes, the identification of the genotype as homozygous or heterozygous was inconclusive at some positions. The Hirisplex [91] and 8-plex [92] prediction systems were used to predict eye, hair and skin color in the ancient samples. Furthermore, we also investigated loci related to lactase persistence.

We used imputation to maximize the genetic information for the phenotypic predictive systems. Imputation allows the inference of missing alleles by comparison of the surrounding haplotypes in the test samples with a phased reference panel [93]. This method has previously been used to infer genotypes from low coverage genomes (∼1x depth of coverage) in ancient specimens, showing a high percentage of concordant predictions when compared to high-coverage observed data [20, 31]. In our analysis, we only considered imputed genotypes if their likelihood score was equal to or higher than 85%.

##### Hair and eye color prediction

The HirisPlex prediction system is an assay based on 24 SNPs used in forensics to predict hair and skin color [91]. Table S3 reports the HirisPlex imputed genotypes in our six ancient samples and Figure S2A reports the probability score of the predicted phenotypes. In cases where incongruences occurred between an imputed and an observed variant, when the observed variant was covered by less than 3 reads and the imputed probability was higher than 0.85, the imputed variant was used for HirisPlex predictions. When the observed variant was covered by less than 3 reads and an imputed probability of less than 0.85, the genotype was left as missing (as, for example, in SC1_Meso rs12913832 and OC1_Meso rs12203592 and rs12913832).

We also investigated pigmentation traits based on the 8-plex system [92], which predicts eye and skin color from the combination of genotypes in eight SNPs, three of which are not included in the HirisPlex system (rs1426654 in gene *SLC24A5*, rs6119471 in gene *ASIP* and rs1545397 in gene *OCA2*, Figure S2B and Table S3). The 8-plex system confirmed the HirisPlex predictions for eye color. GB1_Eneo is homozygous GG at rs12913832, which excludes brown eyes; however, it does not carry any of the combinations of alleles that would determine green or blue eyes based on this test. The most similar combination is GG at rs12913832 and CC at rs16891982, which suggests green eyes, while GB1_Eneo carries GG at rs12913832 and CG at rs16891982. Thus, eye color assignment for GB1_Eneo is inconclusive using 8-plex.

##### Skin color

Based on the 8-plex system, a combination of any two of the following genotypes will result in non-dark skin color: GG at rs12913832, TT at rs1545397, GG at rs16891982, AA at rs1426654, AA at rs885479 and/or TT at rs12203592. Nevertheless, non-white skin color is predicted when GG at rs6119471 occurs together with any of the six alleles for “non-dark skin color.”

The imputed genotypes for these SNPs are included in Table S3. The test was inconclusive for most of our samples as none of them, with the exception of GB1_Eneo, carry any of the possible combinations of alleles proposed by the 8-plex system to determine skin color. However, GB1_Eneo was genotyped as GG at rs12913832 and AA at 1426654, which would predict light skin color in this individual. Moreover, Canes1_Meso was genotype GG at rs12913832, but was heterozygous AG at rs1426654, which is not considered by the 8-plex system.

Despite the inconclusive results, we can still get an indication of skin color if we consider the SNPs included in the 8-plex system, which are located in genes *SLC45A2* and *SLC24A5*: rs16891982 and rs1426654, respectively (Figure S2A). Both genes are related to pigmentation and their distribution in Europe is far from random, as the derived alleles contributing to light skin are almost fixed in modern Europeans. Four of our samples (Chan_Meso, SC1_Meso, SC2_Meso and OC1_Meso) are homozygous for the ancestral allele at these SNP positions: CC at rs16891982 and GG at rs1426654, suggesting that these individuals would not have had light skin. Canes1_Meso is also homozygous CC at rs16891982, but is heterozygous (AG) at rs1426654. GB1_Eneo is heterozygous GC at rs16891982 and homozygous AA at rs1426654. The presence of the derived alleles in these samples could be indicative of lighter skin color in these individuals than in the older samples. Moreover, it shows that by the time of Canes’ population (∼5.3 kya), the alleles determining light skin were already present in the most western part of Europe.

##### Lactase persistence

All ancient samples were homozygous for the ancestral allele (C) at rs182549 (Table S3 and Figure S2A), a genotype that is associated with lactose intolerance in adulthood [94]. Furthermore, except for SC1_Meso, all of them were also homozygous for the ancestral allele (G) at rs4988235, also associated with inability to digest lactose (Table S3 and Figure S2A). SC1_Meso carries an A at this position; however, the genotype could not be determined because the position is covered by only 1 read. Imputation assigned GG as the most likely genotype at this position for SC1_Meso, so it is likely that SC1_Meso was also unable to digest lactose.

#### Mitochondrial analysis and haplogroup assignment

Following whole genome alignment (see S3 for details), we used samtools view to extract the reads mapped to the rCRS to a single bam file. From those bam files, we called positions at which the base differed from that in the reference mitogenome (NC_012920); for this step, we used samtools mpileup and the option -s to specify the ploidy (1). Only bases with quality scores ≥ 30 were considered for analysis. Furthermore, we used Tablet [95] to visualize the alignments and checked by eye all the polymorphic positions reported in the vcf file. Variants called at positions with less than 3 reads of depth, as well as variants called at the last 4 bases of any of the two read ends were excluded from further analysis, as they could have resulted from molecular damage. Haplogroup assignment was based on Haplogrep (http://haplogrep.uibk.ac.at/) (Table S4).

#### Y chromosome analysis

All our three male samples, SC1_Meso, SC2_Meso and OC1_Meso, were assigned to the R1 and R1b haplogroups (Table 1 in main text) using Yfitter, a maximum likelihood method to assign Y haplogroups from low coverage sequence data [43]. Furthermore, we inspected the alignments by eye at defining positions for haplogroup R, following [96] and the updated positions on the ISOGG database. Based on the observed variants, we could confirm the assignment of all samples to haplogroup R1. The individuals SC2_Meso and OC1_Meso were assigned to subhaplogroup R1b1c based on the presence of the following variants:

SC2_Meso: M306(1A), P224(4T), P229(1C), P285(3A), M173(1C), M306(1A), P225(1T), P231(3G), P233(2G), P234(1C), P236(2G), P238(1A), P242(2A), P286(3T), P294(1C), M343(3A), M269(1T
OC1_Meso: M207(1G), M306(1A), P224(2T), P229(1C), P285(2A), M173(1C), M306(1A), P225(2T), P233(1G), P234(1C), P236(2G), P238(1A), P242(1A), P286(1T), M343(2A), M269(1T)

The low coverage of SC1_Meso prevented a more detailed assignment of this individual.

R1b is the major West European linage in present day populations [96]. Ancient DNA studies have reported a notable increase of subhaplogroups R1b and R1a in central Europe during the Late Neolithic and Bronze Age, leading to the hypothesis that they expanded from the East only after ∼4,500 yBP [6], although genetic analysis based on modern populations suggests an older Eastern origin, shortly after the LGM [33]. Recent studies have found haplogroup R1b in a ∼14,000 year old human from Italy [24, 34]) and in a Latvian sample dated to ∼7,000 years [7]. Our Mesolithic samples document the presence of haplogroup R1 in Romania as early as 8,814 ± 261 cal yBP and R1b at 8,703.5 ± 268.5 yBP, which corroborates a wide distribution of the haplogroup in Europe before the Bronze Age.

#### Population genetic analyses

A reference dataset of modern [8, 9] and ancient samples (Data S1) described in [7] was used for population genetic analyses. We also realigned BAM files from [2], as described in the “Processing and alignment of NGS data” section above, and added these samples to our dataset. We also realigned BAM files from [5]. Genotypes in our ancient Romanian and Spanish samples, which overlapped with this dataset, were called using GATK Pileup [38]. Triallelic SNPs were discarded and bases were required to have quality ≥ 30. Alleles were not called within the first and last 2 bp of reads. For positions with more than one base call, one allele was randomly chosen with a probability equal to the frequency of the base at that position. This allele was duplicated to form a homozygous diploid genotype. Only transversion sites were used for PCA, *f*_3_-statistics, *D*-statistics and ADMIXTURE to reduce the effects of post-mortem molecular damage on analyses. Furthermore, ancient samples were required to have ≥15,000 called SNPs in order to meet the inclusion criteria.

Principal components were defined using a Eurasian subset of the modern data and ancient individuals were projected onto the resulting eigenvectors. This analysis was carried out using EIGENSOFT 5.0.1 smartpca [44], removing one SNP from each pair in linkage disequilibrium with *r*^2^ > 0.2 [4] and without removing outlying data.

We used outgroup *f*_3_-statistics [97, 98] to evaluate the amount of shared drift between our ancient samples since their divergence from an African (Mbuti) outgroup. Figure S3A reports the results of these tests for the Spanish samples Chan_Meso and Canes1_Meso, both of which share the most drift with other Western European Hunter-Gatherers. Outgroup *f*_3_ statistics for the Romanian genomes in our dataset are reported in Figure 2 (highest 20 values). *f*_3_-statistics were computed using the qp3Pop program in the ADMIXTOOLS package [44].

*D*-statistics were used to test for admixture [82]. These statistics were computed using the qpDstat programs in the ADMIXTOOLS package [44]. The results of these tests are reported in Table 2.

We used ADMIXTURE [45] to perform a clustering analysis. The minimal cross validation error for the reference dataset has previously been found at 17 clusters (K) [7]. Based on our PCA and *D*-statistics results, we did not expect our Romanian and Spanish samples to define new components, so we added the Romanian and Spanish samples to this dataset and ran 10 runs at K = 17 with fivefold cross-validation and different random seeds. We repeated this analysis at K = 10, K = 15 and K = 20. The results of the ADMIXTURE analysis of all individuals at K = 17 are reported in Figure S3C and for ancient samples at K = 10, K = 15 and K = 20 in Figure S3B.

We used the ADMIXTURE results to estimate hunter-gatherer-, and farmer-related ancestries in our Eneolithic sample GB1_Eneo. We performed bootstrapping by resampling SNPs with replacement 1000 times and estimated errors and confidence intervals on this empirical distribution. For each bootstrap replicate, we inferred the ancestry proportions by numerically maximizing the following likelihood function on the bootstrapped SNPs, following the logic of [22, 99]:

$$\text{L}\left(\text{Q},\text{F}\right)=\sum_{\text{i}}\sum_{\text{j}}\left\{{\text{g}}_{\text{ij}}\text{ln}\left[\sum_{\text{k}}{\text{q}}_{\text{ik}}{\text{f}}_{\text{kj}}\right]+\left(2-{\text{g}}_{\text{ij}}\right)\text{ln}\left[\sum_{\text{k}}{\text{q}}_{\text{ik}}\left(1-{\text{f}}_{\text{kj}}\right)\right]\right\},$$

where g_ij_ is the count of allele 1 (0, 1 or 2) of individual i at SNP j, q_ik_ is the fraction the inferred ancestral population k contributes to individual i’s genome and f_kj_ is the frequency of allele 1 at SNP j in the inferred ancestral population k. The likelihood was maximized using the minimize function from scipy, with the “L-BFGS-B” method and proportions bounded between [1e-5,1-1e-5] and constrained to sum to unity. We used inferred allele frequencies of the ancestral populations, f_kj_, from the run at K = 17 with the lowest cross-validation error.

We used a custom python script to perform bootstrapping on a single individual. The code is available on GitHub: https://github.com/siskavera/genetics_scripts/tree/master/admixture-projection.

We examined runs of homozygosity in our two samples with the highest coverage: GB1_Eneo and Chan_Meso. We also included published high coverage hunter-gatherer and Neolithic farmer samples for comparison (namely NE1 [32], Bichon [20], Stuttgart and Loschbour [8] and Bon002 [2]). We trimmed 10 bp from read termini to mitigate the effects of postmortem damage which tends to be concentrated at the ends of reads. We called genotypes in our samples at positions which had a minor allele frequency of ≥ 10% in Yoruban individuals from Phase 3 of the 1000 Genomes Project [2, 100] using GATK [51]. Genotypes in our ancient samples were required to have a minimum depth of 4, a maximum depth of twice the average genome coverage and heterozygous sites were required to have each allele called at least twice. ROH analysis which was carried out using PLINK [46] following the parameters described in [32].

We estimated the proportion of Neanderthal ancestry (Q) in each of our samples using the *f*_4_ ratio described in [24]:

$$Q=1-\frac{f_{4}\left(West\;and\;Central\;Africans,\;Chimp;test,\;Altai\;Neanderthal\right)}{f_{4}\left(West\;and\;Central\;Africans,\;Chimp;Dinka,Altai\;Neanderthal\right)}.$$

We extracted genotypes from Dinka, West and Central African (i.e., Mbuti, Yoruba and Mende), Chimp and Altai Neanderthal samples which were included in the Simons Diversity Project [101]. We kept sites which were biallelic in Phase 3 of the 1000 Genomes Project [100] and which overlapped the 2.2 million SNP capture panel described in [24]. For each of our ancient samples we clipped 10bp from the starts and ends of reads for the reasons described above. We called pseudo-haploid genotypes in our samples as described at the beginning of this section. We merged these data with the Simons Panel data using PLINK [46] and calculated f4 ratios using the qpF4ratio package in ADMIXTOOLS [44]. For context in Figure S4B we also include estimates of Neanderthal ancestry for a selection of Late Pleistocene and Holocene samples (those included in [24] Figure 2 but with Oase1 omitted as it has recent Neanderthal introgression).

### Data and Software Availability

All newly generated genome data have been deposited in ENA: PRJEB20614 and PRJEB20616.

## Author Contributions

M.H., A.M., and R.P., supervised the study. G.G.-F. performed sampling and labwork. G.G.-F., E.R.J., V.S., and A.M. analyzed genetic data. E.L. and C.B. performed isotope analysis. E.L., A.G.-D., and C.B. analyzed and/or helped to interpret isotope data. R.P., C.B., C.L., A.G.-D., M.D.G., L.D., A.S., A.B., J.R.V.R., M.V.R., and P.A. provided samples and/or input about archaeological context. G.G.-F., E.R.J., A.M., C.B., and M.H. wrote the manuscript with input from all co-authors.

## Supplemental Information

Document S1. Figures S1–S4 and Tables S1–S4

Data S1. Dataset of Ancient Samples Used for Population Genetic Analyses, Related to Figures 1, 2, and S3 and STAR Methods

Data S2. Results of the Outgroup *f*_*3*_ Statistics for the Romanian Genomes, Related to Figures 2 and S3 and STAR Methods

Document S2. Article plus Supplemental Information
