# Supplementary material for: Paleogenomic Evidence for Multi-generational Mixing between Neolithic Farmers and Mesolithic Hunter-Gatherers in the Lower Danube Basin
Source: Curr Biol. 2017 Jun 19;27(12):1801–1810.e10. doi: 10.1016/j.cub.2017.05.023 (PMC5483232; doi:10.1016/j.cub.2017.05.023)
Supplement: Document S1. Figures S1–S4 and Tables S1–S4 [file mmc1.pdf]

Current Biology, Volume *27*

Supplemental Information

Paleogenomic Evidence for Multi-generational Mixing between Neolithic Farmers and Mesolithic Hunter-Gatherers in the Lower Danube Basin

Gloria González-Fortes, Eppie R. Jones, Emma Lightfoot, Clive Bonsall, Catalin Lazar, Aurora Grandal-d'Anglade, María Dolores Garralda, Labib Drak, Veronika Siska, Angela Simalcsik, Adina Boroneanţ, Juan Ramón Vidal Romaní, Marcos Vaqueiro Rodríguez, Pablo Arias, Ron Pinhasi, Andrea Manica, and Michael Hofreiter

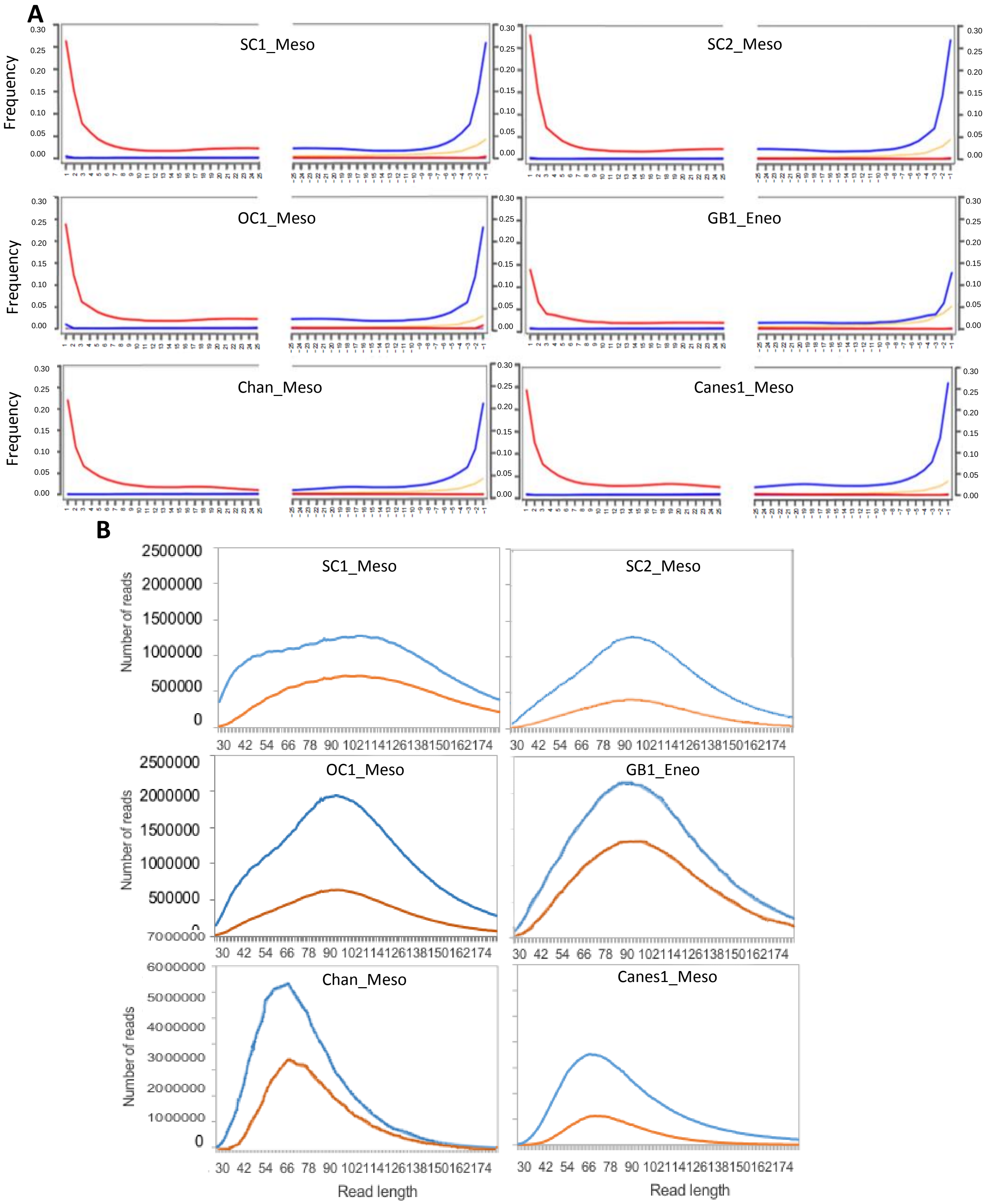

Figure S1. **Patterns of molecular damage in aDNA sequences**. Related to Figure 1. **A.** Deamination patterns at the edges of the NGS reads estimated by mapDamage for each of the six ancient human samples. Graphs on the left show the C to T deamination rate at the 5' end (in red), and on the right the G to A rate at the 3' end (in blue). **B.** Read length distributions of the DNA sequences. For each sample, the read length distribution of all reads (mapped and unmapped) is given in blue and the read length distribution of mapped reads in orange.

A

Gene	*SLC24A5*	*SLC45A2*	*MCM*		Eye color	Hair color	
SNP identifier	rs1426654	rs16891982	rs182549	rs4988235		Color	Shade
SC1_Meso					Brown (0.952)	Black (0.65)	Dark (0.84)
SC2_Meso	* *	* *	* *	* *	Brown (0.976)	Black (0.908)	Dark (0.997)
OC1_Meso	* *	* *	* *	* *	Brown (0.988)	Black (0.753)	Dark (0.997)
GB1_Eneo	* *	* *		* *	Blue (0.783)	Black (0.432)	Dark (0.686)
Chan_Meso	* *	* *	* *	* *	Brown (0.952)	Black (0.831)	Dark (0.986)
Canes1_Meso	* *	* *			Blue (0.588)	Black (0.708)	Dark (0.963)

ancestral allele derived allele

B

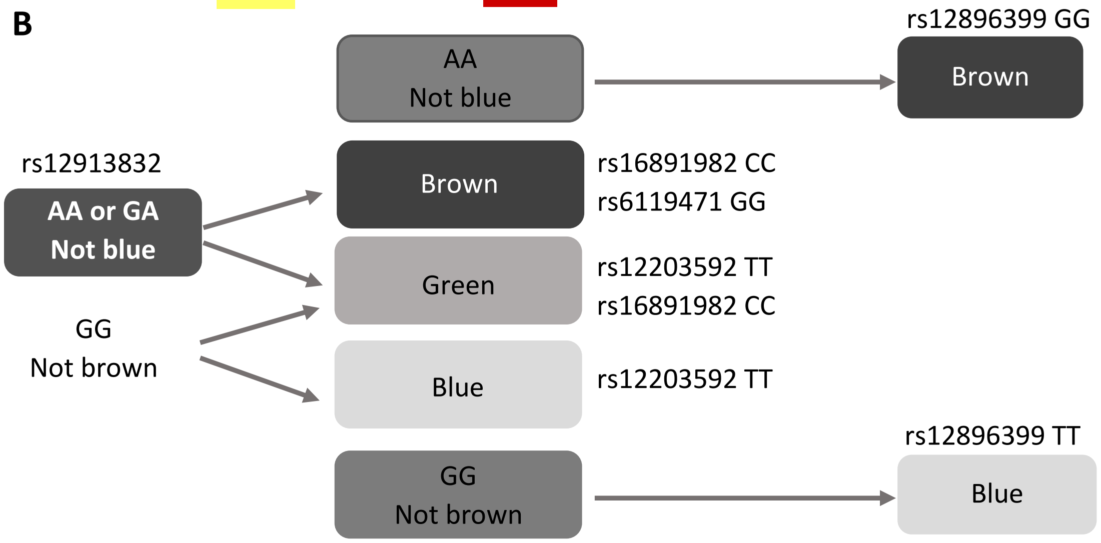

Figure S2. Imputed genotypes of interest, along with phenotypic predictions based on the HirisPlex and 8-Plex prediction systems A. Related to Figure 1. Imputed genotypes for skin color and lactose tolerance in the ancient Spanish and Romanian samples, together with their hair and eye color predicted phenotypes based on the HirisPlex prediction system. The asterisks indicate that the imputed genotype is supported by observed data with at least 3 reads of coverage. **B.** Diagram for eye color phenotype assignment based on the 8-plex prediction system (modified from [S1]).

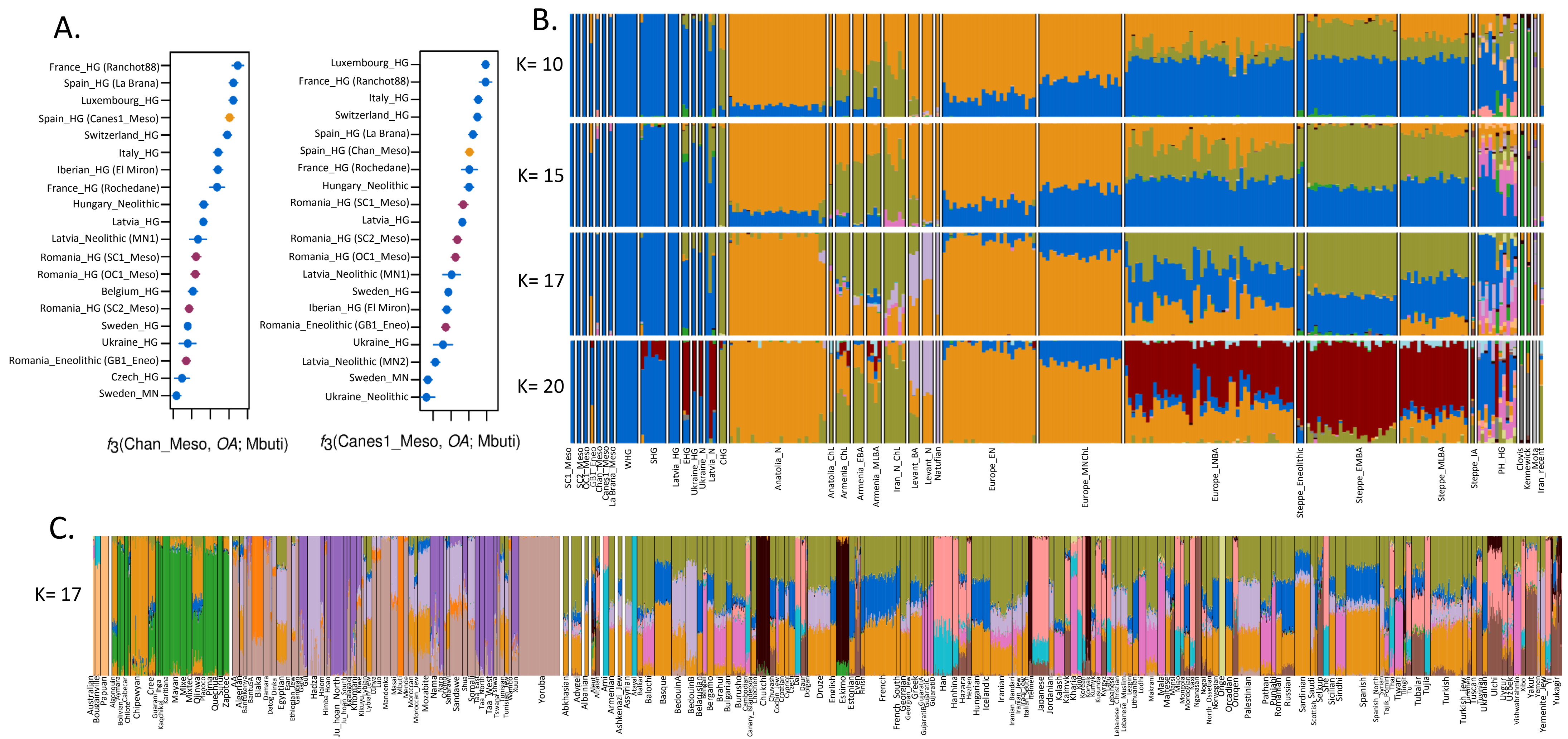

Figure S3. Outgroup f3-statistics and ADMIXTURE analysis. Related to Figure 1 and Figure 2. A. Outgroup f3-statistics of the form f3(Spanish Mesolithic, *other ancient (OA)*; Mbuti). For each test, the highest 20 values are shown. The Spanish Mesolithic samples, Chan_Meso and Canes1_Meso, share the most drift with other hunter-gatherer samples from Western Europe (Ranchot88, La Braña, Loschbour, Bichon, and each other), followed by the Hungarian HG, Latvian HG and the Romanian Mesolithics. **B**. ADMIXTURE analysis for all ancient individuals at K=10, K=15, K=17 and K=20. **C**. ADMIXTURE analysis for modern individuals at K=17.

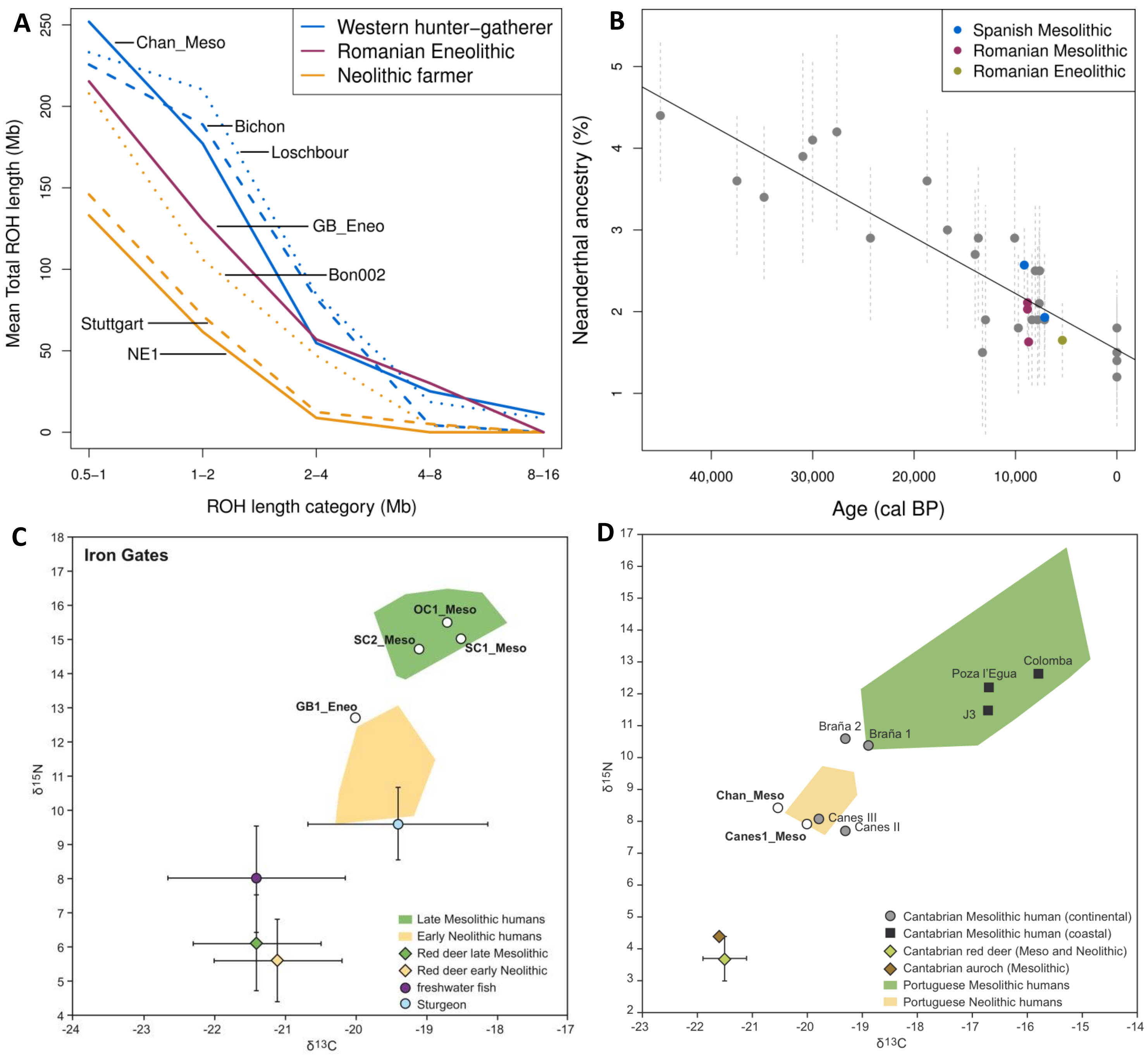

Figure S4. Runs of Homozygosity, estimate of the proportion of Neanderthal ancestry and bivariate plots of stable isotope values. A. Runs of Homozygosity in the samples with highest genome coverage (Chan1_Meso and GB1_Eneo). Related to Figure 1. Published high coverage hunter-gatherer (Bichon and Louschbour) and Neolithic farmer (NE1, Stuttgart and Bon002) samples were included for comparison. B. Estimate of the proportion of Neanderthal ancestry in our ancient samples. C and D. Bivariate plot of stable isotopic values of human remains and associated fauna. In C Romanian human remains (this paper), Late Mesolithic and Early Neolithic Iron Gates humans [S2, S3, S4], freshwater and marine fishes [S5] and coeval red deer (Bonsall, unpublished). In D Chan_Meso human and associated aurochs [S6], Canes1_Meso [S7], Mesolithic Cantabrian humans [S7, S8], Mesolithic and Neolithic Portuguese humans [S9], and Mesolithic and Neolithic Cantabrian red deer (n=14) from El Mirón Cave [S10]. Error bars for the aurochs (n=3) are smaller than the symbol. Related to Figure 1 and Table 1.

.

Table S1. Radiocarbon dates of the ancient samples analyzed in this study along with their percentage (%) of human DNA estimated from the MiSeq run. Related to Table 1.

Archaeological ID	aDNA laboratory ID[a]	Site	Lab ID	^{14}C age BP	cal BP age (± 1σ) [OxCal 4.2]	cal BP age range (2σ) [OxCal 4.2]	% human DNA
M95/2	SC1_Meso	Schela Cladovei (Romania)	OxA-8583	8,380 ± 80[b]	8,817 ± 135	9,075-8,553	30.29
M96/3	SC2_Meso	Schela Cladovei (Romania)	-	-	-	-	32.95
M24	OC1_Meso	Ostrovul Corbului (Romania)	MAMS-28615	8,277 ± 34[b]	8,644 ± 117	8,972-8,435	47.42
M1	GB1_Eneo	Gura Baciului (Romania)	MAMS-28614	4,621 ± 28	5,388 ± 54	5,456-5,299	65.37
Elba	Chan_Meso	Chan do Lindeiro (Spain)	Ua-13398 /38115	8,155 ± 42[c]	9,106 ± 68	9,255-9,007	52.22
I-A	Canes1_Meso	Canes (Spain)	OxA-7148 /AA-5294	6,197 ± 45[c]	7,096 ± 69	7,245-6,985	33.79
II-A	Canes2	Canes (Spain)	AA-5296 /11744 AA-11744/OxA 23185	7,092± 31[c]	7,916 ± 44	7,974-7,850	11.60
	eBLd						0.25
	liBLd						0.90
	eBLr						0.40
	liBLr						0.42

[a] The blanks are identified as eBL for the extraction and liBL for the library building processes. Suffix d and r refers to the DNA extraction protocol followed for the corresponding samples (d: [S11]; r: [S12], respectively).
[b] ^{14}C dates from Iron Gates must be corrected considering FRE, which results in 7960 ± 96 ^{14}C age BP for SC1_Meso and 7826 ± 67 age BP for OC1_Meso.
[c] Weighted mean calculated by r_combine in Oxcal 4.2 (INTCAL13 curve).

Table S2. Summary of reads processed from HiSeq sequencing and estimates of contamination based on mitochondrial (mt) and X chromosome (X) sequences. Related to Table 1.

Sample ID	Total reads	Mapped not clonal reads with q ≥ 30	Human DNA (%)	Average depth of coverage	mt depth of coverage	mt Contamin. (c+md/c-md)[a]	X Contamin. (Test1/Test2)	Ry[b]
**SC1_Meso**	118,983,040	31,452,424	26.43	1.11x	40.95x	1.51/1.15	1.02±0.2/0.84±0.3	0.095±8.3E-08
**SC2_Meso**	174,295,430	74,180,397	42.56	2.83x	137.80x	1.27/1.02	1.01±0.1/1.13±0.1	0.098±1.89E-07
**OC1_Meso**	193,724,173	52,424,548	27.06	1.86x	76.53x	1.32/0.68	2.21±0.1/2.18±0.3	0.095±1.14E-07
**GB1_Eneo**	204,276,912	112,873,035	55.25	4.05x	174.43x	0.43/0.32	NA	0.004±1.38E-09
**Chan_Meso**	354,735,644	177,566,984	50.05	5.28x	178.42x	1.30/1.24	NA	0.004±1.02E-09
**Canes1_Meso**	246,349,872	58,518,449	23.75	1.73x	67.38x	1.41/1.15	NA	0.0046±3.67E-09

[a](C + MD), percentage contamination including sites with potentially damaged bases. (C - MD), percentage of contamination excluding sites with potentially damaged bases (C to T and G to A transitions).
[b]Ry: rate of reads aligning to the Y chromosome compared to the total number of reads aligning to the sex chromosomes.

Table S3. Imputed genotypes for the SNP panel used in the HirisPlex system, 8-plex and genes related with lactose tolerance in adulthood. Related to Figure 1.

		**allele**		**SC1_Meso**		**SC2_Meso**		**OC_Meso**		**GB_Eneo**		**Chan_Meso**		**Canes**	
	**SNP identifier**	**0**	**1**	**gen**	**P**	**gen**	**P**	**gen**	**P**	**gen**	**P**	**gen**	**P**	**gen**	**P**
MC1R	rs11547464	G	A	0/0	0.999	0.0	0.999	0/0	0.995	0/0	0.92	0/0	1	0/0	1
MC1R	rs1805008	C	T	0/0	1	0/0	1	0/0	1	0/0	1	0/0	1	0/0	1
MC1R	rs1805005	G	T	0/0	0.989	0/0	0.961	0/0	0.877	0/0	0.999	0/0	1	0/0	1
MC1R	rs1805006	C	A	0/0	1	0/0	1	0/0	1	0/0	1	0/0	1	0/0	1
MC1R	rs1805007	C	T	0/0	0.949	0/0	0.998	0/0	0.537	0/0	0.999	0/0	1	0/0	1
MC1R	rs1805009	G	C	0/0	0.952	0/01	1	0/0	1	0/0	1	0/0	1	0/0	1
MC1R	rs2228479	G	A	0/0	0/998	0/0	1	0/0	1	0/0	1	0/0	1	0/0	1
MC1R	rs1110400	T	C	0/0	1	0/0	1	0/0	1	0/0	1	0/0	1	0/0	1
SLC45A2	rs28777	C	A	0./1	0.761	1/1	1	0/0	0.997	0/1	1	0/0	1	0/0	1
KITLG	rs12821256	T	C	0/0	1	0/0	1	0/0	0.999	0/0	1	0/0	1	0/0	0.968
EXOC2	rs4959270	C	A	1/1	0.998	1/1	1	0/0	1	1/1	1	0/0	1	1/1	1
TYR	rs1042602	C	A	0/0	1	0/0	1	0/0	1	0/0	1	0/0	1	0/0	1
OCA2	rs1800407	C	T	0/0	1	0/0	1	0/0	0.999	0/0	1	0/0	1	0/0	1
SLC24A4	rs2402130	G	A	1/1	1	1/1	1	0/1	1	1/1	1	1/1	0.999	0/1	0.983
ASIP/PIGU	rs2378249	G	A	0/1	1	1/1	1	0/1	1	0/1	1	1/1	1	0/1	1
TYR	rs1393350	G	A	0/0	1	0/0	1	0/0	1	0/1	0.822	0/0	1	0/0	0.967
TYRP1	rs683	C	A	1/1	1	1/1	1	1/1	1	1/1	1	0/0	1	1/1	0.999
SLC45A2	**rs16891982**	C	G	0/0	0.92	0/0	0.986	0/0	1	0/1	1	0/0	1	0/0	1
MC1R	**rs885479**	G	A	0/0	0.888	0/0	0.997	0/0	1	0/0	1	0/0	1	0/0	1
IRF4	**rs12203592**	C	T	0/0	0.862	1/1	0.554	0/1	0.623	0/1	0.69	0/0	1	0/1	0.757
HERC2	**rs12913832**	A	G	0/1	0.802	0/0	1	0/1	0.56	1/1	0.521	0/1	0.989	1/1	0.94
SLC24A4	**rs12896399**	G	T	0/0	1	0/0	1	0/1	1	0/0	1	0/0	0.999	0/1	0.994
OCA2	**rs1545397**	A	T	0/0	0.994	0/0	0.999	0/0	0.978	0/0	1	0/0	0.996	0/0	1
SLC24A5	**rs1426654**	A	G	1/1	0.93	1/1	1	1/1	0.987	0/0	1	1/1	1	0/1	0.998
ASIP	**rs6119471**	C	G	0/0	1	0/0	1	0/0	1	0/0	1	0/0	1	0/0	1
MCM6	rs4988235	G	A	0/0	1	0/0	1	0/0	1	0/0	1	0/0	1	0/0	1
MCM6	rs182549	C	T	0/0	1	0/0	1	0/0	1	0/0	1	0/0	1	0/0	1

gen: genotype; P: probability

In bold: diagnostic SNPs for the 8-plex system.

The SNPs at the *MCM6* gene are associated with lactose tolerance in adulthood.

Table S4. Mitochondrial haplogroups and haplotypes for the studied samples. Related to Table 1.

Sample ID	Coverage	Haplogroup	Haplotype		
			Defining mutations	Mutations expected but not found*	Other variants
SC1_Meso	40.95x	U5b2c	73G, 150T, 263G, 723G, 750G, 1438G, 1721T, 2706G, 3197C, 7028T, 7768G, 9477A, 11467G, 11719A, 12308G, 12372A, 13017G, 13617C, 13637G, 14182C, 14766T, 15326G, 16192T, 16270T	960.XC, 4769G, 8860A	236C, 16278T
SC2_Meso	137.80x	U5a1c	73G, 263G, 750G, 1438G, 2706G, 3197C, 4769G, 7028T, 9477A, 11467G, 11719A, 12308G, 12372A, 13617C, 14766T, 14793G, 15218G, 15326G, 16192T, 16256T, 16270T, 16320T, 16399G	8860A	7080C, 14212C
OC1_Meso	76.53x	K1 + 16362	73G, 263G, 750G, 1189C, 1438G, 1811G, 2706G, 3480G, 7028T, 9055A, 9698C, 10398G, 10550G, 11299C, 11467G, 11719A, 12308G, 12372A, 14167T, 14766T, 14798C, 15326G, 16224C, 16311C, 16362C	4769G, 8860A	152C, 12130C, 16519C
GB1_Eneo	174.43x	K1a4a	73G, 263G, 497T, 750G, 1189C, 1438G, 1811G, 2706G, 3480G, 4769G, 6260A, 7028T, 9055A, 9698C, 10398G, 10550G, 11299C, 11467G, 11485C, 11719A, 12308G, 12372A, 14167T, 14766T, 14798C, 15326G, 16224C, 16311C	8860A, 16093C	16168T, 16519C
Chan_Meso	178.42x	U5b	73G, 150T, 263G, 750G, 1438G, 2706G, 3197C, 7028T, 7768G, 9477A, 11467G, 11719A, 12308G, 12372A, 13617C, 14182C, 14766T, 15326G, 16192T, 16270T	4769G, 8860A	6713T

Canes1_Meso	67.38x	U5a2a	73G, 263G, 750G, 1438G, 2706G, 3197C, 9477A, 11467G, 11719A, 12308G, 12372A, 13617C, 14766T, 14793G, 16114A, 16192T, 16256T, 16270T, 16294T, 16526A	4769G, 7028T, 8860A

* After checking the alignments for the expected but not covered mutations, we could confirm that all but one (16093C in GB1_Eneo which was monomorphic T) were positions not called for SNP identification because the coverage was too low (less than 3) or because they were only covered by bases called within 4 bp of the ends of the reads

SUPPLEMENTAL REFERENCES:

S1. Hart, K.L., Kimura, S.L., Mushailov, V., Budimlija, Z.M., Prinz, M., and Wurmbach E. (2013). Improved eye- and skin-color prediction based on 8 SNPs. Croat Med J. *54*, 248–256.

S2. Borić, D., Grupe, G., Peters, J., and Mikić, Z. (2004). Is the Mesolithic–Neolithic subsistence dichotomy real? New stable isotope evidence from the Danube Gorges. J. Eur. Archaeol. *7*, 221–248.

S3. Bonsall, C., Cook, G., Pickard, C., McSweeney, K., Sayle, K., Bartosiewicz, L., Radovanović, I., Higham, T., Soficaru, A., and Boroneant, A. (2015). Food for thought: re-assessing Mesolithic diets in the Iron Gates. Radiocarbon *57*, 689–699.

S4. Bonsall, C., Boroneanț, A., Simalcsik, A., and Higham, T. (2016). Radiocarbon dating of Mesolithic burials from Ostrovul Corbului, southwest Romania. In Southeast Europe and Anatolia in Prehistory. Essays in Honor of Vassil Nikolov on his 65th Anniversary, K. Bacvarov and R. Gleser, eds (Universitätsforschungen zur Prähistorischen Archäologie 293. Bonn, Habelt) pp. 41–50.

S5. Bonsall, C., Cook, G.T., Hedges, R.E.M., Higham, T.G.F., Pickard, C., and Radovanovic, I. (2004). Radiocarbon and stable isotope evidence of dietary change from the Mesolithic to the Middles Ages in the Iron Gates: new results from Lepenski Vir. Radiocarbon *46*, 293–300.

S6. Grandal d'Anglade, A. and Vidal Gorosquieta, A. (2017). Caracterización isotópica de Elba, la mujer mesolítica de Chan do Lindeiro (Pedrafita, Lugo, Península Ibérica). Cadernos do Laboratorio Xeolóxico de Laxe *39*, 89–110.

S7. Arias, P. (2005). Determinaciones de isótopos estables en restos humanos de la región Cantábrica. Aportación al estudio de la dieta de las poblaciones del Mesolítico y el Neolítico. Munibe, *57*, pp. 359–374.

S8. Arias, P., and Schulting, R.J. (2010). Análisis de isótopos estables sobre los restos humanos de La Braña-Arintero. Aproximación a la dieta de los grupos mesolíticos de la cordillera cantábrica. In Los hombres mesolíticos de la cueva de La Braña-Arintero (Valdelugueros, León), Vidal J. and Prada M.E., ed. (Consejería de Cultura y Turismo, Junta de Castilla y León. León), pp. 129–137.

S9. Lubell, D., Jackes, M., Schwarcz, H., Knyf, M., and Meiklejohn, C. (1994). The Mesolithic-Neolithic transition in Portugal: isotopic and dental evidence of diet. Journal of Archaeological Science *21*, 201–216.

S10. Stevens, R.E., Hermoso-Buxán, X.L., Marín-Arroyo, A.B., González-Morales, M.R., and Straus, L.G. (2014). Investigation of Late Pleistocene and Early Holocene palaeoenvironmental change at El Mirón cave (Cantabria, Spain): Insights from carbon and nitrogen isotope analyses of red deer. Palaeogeography, Palaeoclimatology, Palaeoecology *414*, 46–60.

S11. Dabney, J., Knapp, M., Glocke, J., Gansauge, M.T., Weihmann, A., Nickel, B., Valdiosera, C., García, N., Pääbo, S., Arsuaga, J.L., et al. (2013). Complete mitochondrial genome sequence of a Middle Pleistocene cave bear reconstructed from ultrashort DNA fragments. PNAS *110*, 15758–15763.

S12. Rohland, N., Siedel, H., and Hofreiter, M. (2010). A rapid column-based ancient DNA extraction method for increased sample throughput. Mol Ecol Resour *10*, 677–683.

S13. Fu, Q., Posth, C., Hajdinjak, M., Petr, M., Mallick, S., Fernandes, D., Furtwängler, A., Haak, W., Meyer, M., and Mittnik, A. (2016). The genetic history of Ice Age Europe. Nature 534, 200–205.

S14. Lazaridis, I., Nadel, D., Rollefson, G., Merrett, D.C., Rohland, N., Mallick, S., Fernandes, D., Novak, M., Gamarra, B., Sirak, K., et al. (2016). Genomic insights into the origin of farming in the ancient Near East. Nature *536*, 419–424.

S15. Mathieson, I., Lazaridis, I., Rohland, N., Mallick, S., Patterson, N., Roodenberg, S.A., Harney, E., Stewardson, K., Fernandes, D., Novak, M., et al. (2015). Genome-wide patterns of selection in 230 ancient Eurasians. Nature *528*, 499–503.

S16. Rasmussen, M., Anzick, S., Waters, M.R., Skoglund, P., DeGiorgio, M., Stafford T.W., Rasmussen, S., Moltke, I., Albrechtsen, A., Doyle, S.M., et al. (2014). The genome of a Late Pleistocene human from a Clovis burial site in western Montana. Nature *506*, 225-229

S17. Allentoft, M.E., Sikora, M., Sjogren, K.-G., Rasmussen, S., Rasmussen, M., Stenderup, J., Damgaard, P.B., Schroeder, H., Ahlström, T., Vinner, L., et al. (2015). Population genomics of Bronze Age Eurasia. Nature *522*, 167–172.

S18. Jones, E.R., Gonzalez-Fortes, G., Connell, S., Siska, V., Eriksson, A., Martiniano, R., McLaughlin, R.L., Gallego Llorente, M., Cassidy, L.M., Gamba, C., et al. (2015). Upper Palaeolithic genomes reveal deep roots of modern Eurasians. Nat. Commun. *6*, 8912.

S19. Haak, W., Lazaridis, I., Patterson, N., Rohland, N., Mallick, S., Llamas, B., Brandt, G., Nordenfelt, S., Harney, E., Stewardson, K., et al. (2015). Massive migration from the steppe was a source for Indo-European languages in Europe. Nature *522*, 207–211.

S20. Olalde, I., Allentoft, M.E., Sánchez-Quinto, F., Santpere, G., Chiang, CW., DeGiorgio, M., Prado-Martinez, J., Rodríguez, JA., Rasmussen, S., Quilez, J., et al. (2014). Derived immune and ancestral pigmentation alleles in a 7,000-year-old Mesolithic European. Nature *507*, 225–228.

S21. Gamba, C., Jones, E.R., Teasdale, M.D., McLaughlin, R.L., Gonzalez-Fortes, G., Mattiangeli, V., Domboroczki, L., Kovari, I., Pap, I., Anders, A., et al. (2014). Genome flux and stasis in a five millennium transect of European prehistory. Nat. Commun. *5*, 5257.

S22. Lazaridis, I., Patterson, N., Mittnik, A., Renaud, G., Mallick, S., Kirsanow, K., Sudmant, PH., Schraiber, JG., Castellano, S., Lipson, M., et al. (2014). Ancient human genomes suggest three ancestral populations for present-day Europeans. Nature *513*, 409–413.

S23. Günther T., Valdiosera, C., Malmström H., Ureña, I., Rodriguez-Varela R., Sverrisdóttir, O.O., Daskalaki E.A., Skoglund, P., Naidoo, T., Svensson, E.M., et al. (2015). Ancient genomes link early farmers from Atapuerca in Spain to modern-day Basques. PNAS *112*, 11917–11922.

S24. Cassidy, L.M., Martiniano, R., Murphy, E.M., Teasdale, M., Mallory, J., Hartwell, B., Bradley, D.G. (2016). Neolithic and Bronze Age migration to Ireland and establishment of the insular Atlantic genome. PNAS *113*, 368–373

S25. Rasmussen, M., Sikora, M., Albrechtsen, A., Korneliussen, T.S., Moreno-Mayar, J.V., Poznik, G.D., Zollikofer, C.P.E., Ponce de León, M.S., Allentoft, M.E., Moltke, I., et al. (2015). The ancestry and affiliations of Kennewick Man. Nature *523*, 455–458

S26. Seguin-Orlando, A., Korneliussen, T.S., Sikora, M., Malaspinas, A.S., Manica, A., Moltke, I., Albrechtsen, A., Ko, A., Margaryan, A., Moiseyev, T.G. (2014). Genomic structure in Europeans dating back at least 36,200 years. Science *346*, 1113–1118

S27. Raghavan, M., Skoglund, P., Graf, K.E., Metspalu, M., Albrechtsen, A., Moltke, I., Rasmussen, S., Stafford, T.W. Jr., Orlando, L., Metspalu, E., et al. (2014). Upper Palaeolithic Siberian genome reveals dual ancestry of Native Americans. Nature 505:87–91

S28. Gallego Llorente, M., Jones, E.R., Eriksson, A., Siska, V., Arthur, K.W., Arthur, J.W., Curtis, M.C., Stock, J.T., Coltorti, M., Pieruccini, P., et al. (2015). Ancient Ethiopian genome reveals extensive Eurasian admixture throughout the African continent. Science *350*, 820-822.

S29. Fu, Q., Hajdinjak, M., Moldovan, O.T., Constantin, S., Mallick, S., Skoglund, P., Patterson, N., Rohland, N., Lazaridis, I., Nickel, B., et al. (2015). An early modern human from Romania with a recent Neanderthal ancestor. Nature *524*, 216–219.

S30. Fu, Q., Li, H., Moorjani, P., Jay, F., Slepchenko, S.M., Bondarev, A.A., Johnson, P.L., Aximu-Petri, A., Prüfer, K., Filippo, C., et al. (2014). Genome sequence of a 45,000-year-old modern human from western Siberia. Nature *514*, 445–449.

S31. Skoglund, P., Malmström, H., Omrak, A., Raghavan, M., Valdiosera, C., Günther, T., Hall, P., Tambets, K., Parik, J., Sjögren, KG., et al. (2014). Genomic diversity and admixture differs for Stone-Age Scandinavian foragers and farmers. Science *344*, 747–750.

S32. Kılınç, G.M., Omrak, A., Özer, F., Günther, T., Büyükkarakaya, A.M., Biçakçi, E., Baird, H., Dönertaş, H.M., Ghalichi, A., et al. (2016). The demographic development of the first farmers in Anatolia. Current Biology *19*, 2659–2666.

S33. Jones, E.R., Zarina, G., Moiseyev V., Lightfoot, E., Nigst, P., Manica, A., Pinhasi, R., Bradley, D. et al. (2017). The Neolithic transition in the Baltic was not driven by admixture with early European farmers. Current Biology. doi: 10.1016/j.cub.2016.12.060
